# Epigenetic–metabolic coupling by SOX3/WDR5-SIRT5 signaling mediates the therapeutic effects of TGFβ-targeted exosomal nanoparticles in osteoarthritis

**DOI:** 10.1016/j.mtbio.2026.103457

**Published:** 2026-07-21

**Authors:** Fu Qiwei, Shao Jiahua, Chen Shu, Cao Jia, Li Haobo, Zhu Jun

**Affiliations:** Department of Orthopedics, Shanghai Changzheng Hospital, Naval Medical University, No.415 Fengyang Road, Shanghai, 200003, PR China

**Keywords:** Exosome, Osteoarthritis, SOX3, H3K4me3, Cell death, Epigenetic modification

## Abstract

The development of targeted nanotherapeutics capable of restoring chondrocyte homeostasis represents a major challenge in osteoarthritis (OA) treatment. In this study, we engineered a transforming growth factor-β (TGF-β) receptor–targeted exosome–nanoparticle hybrid system (TGFβ-Exo-NP) derived from chondrocytes and systematically evaluated its therapeutic efficacy and molecular mechanism. TGFβ-Exo-NP displayed uniform nanoscale morphology, favorable surface charge, and controlled release properties, with robust chondrocyte-specific uptake mediated by transforming growth factor-β receptor (TGF-βR) targeting. Functionally, TGFβ-Exo-NP markedly restored mitochondrial function, increased adenosine triphosphate (ATP) production, and inhibited apoptosis and pyroptosis in IL-1β–challenged chondrocytes, while promoting anabolic extracellular matrix synthesis. Integrated ChIP-seq, RNA-seq, and proteomic analyses demonstrated that TGFβ-Exo-NP activated a SOX3-driven epigenetic program involving histone H3 lysine 4 trimethylation (H3K4me3) enrichment and SIRT5 upregulation, thereby reprogramming mitochondrial metabolism. WDR5 was identified as a key exosomal effector protein required for chromatin activation and metabolic recovery. *In vivo*, TGFβ-Exo-NP significantly ameliorated cartilage destruction and subchondral bone remodeling in a destabilization of the medial meniscus (DMM) rat model, with improved gait performance and histological outcomes. These results establish a multifunctional exosome-based nanosystem as a precision therapeutic strategy and provide mechanistic insights into epigenetic–metabolic reprogramming in OA.

## Graphical abstract

TGFβ-Exo-NP Attenuates Cell Death-Related Pathways in OA by Activating the SOX3-H3K4me3 Signaling Axis.

**Figure:**
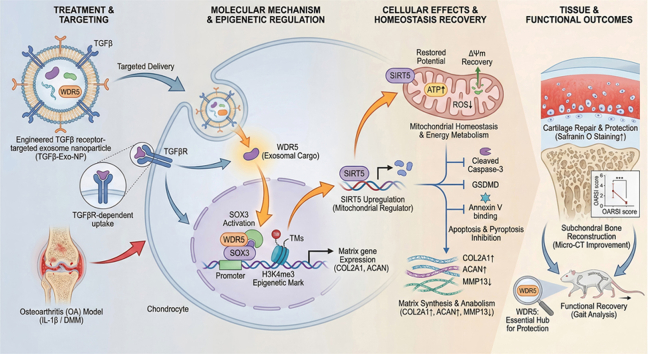

## 1 Introduction

Osteoarthritis (OA) is a degenerative disease characterized primarily by cartilage degradation, subchondral bone remodeling, and chronic inflammation, severely impairing quality of life in middle-aged and elderly populations [1,2]. With the global trend of population aging, the incidence of OA has continued to rise, making it one of the leading causes of disability worldwide [3]. Current clinical treatments mainly focus on pain relief, anti-inflammatory therapy, and maintenance of joint function, but fail to halt disease progression at its root [4]. More critically, there are no approved disease-modifying OA drugs capable of effectively reversing cartilage degeneration [5]. Therefore, a deeper understanding of OA pathogenesis—particularly the identification of cellular-level therapeutic targets and strategies for precision intervention—has become a focal point of both basic and translational OA research.

During OA development, chondrocyte dysfunction and various forms of programmed cell death, including apoptosis, pyroptosis, and ferroptosis, are closely involved. These death pathways are co-activated in the OA microenvironment, collectively accelerating cartilage matrix degradation and tissue structural damage [6,7]. Studies have shown that mitochondria, as the central hubs of cellular energy metabolism, exhibit marked dysfunction in OA, evidenced by membrane potential collapse, decreased adenosine triphosphate (ATP) production, and elevated oxidative stress [8]. Notably, downregulation of the mitochondrial deacetylase SIRT5 has been shown to induce metabolic imbalance and promote chondrocyte apoptosis [9]. In addition, OA-associated chondrocytes undergo energy metabolic reprogramming, suggesting that targeting mitochondrial homeostasis may represent a promising metabolic intervention strategy [10,11]. Achieving coordinated regulation of metabolic restoration and cellular protection remains a critical scientific challenge in OA therapy.

In recent years, exosome-based nanoscale delivery systems have attracted increasing attention for targeted disease therapy due to their excellent biocompatibility, tissue-penetrating capabilities, and programmability [12]. Exosomes can stably carry and deliver functional molecules such as proteins and nucleic acids, participate in intercellular communication, and maintain tissue homeostasis, making them uniquely advantageous as therapeutic carriers [[13], [14], [15]]. Simultaneously, epigenetic mechanisms have attracted growing interest in OA research, particularly the role of histone methylation in regulating gene transcription [16,17]. SOX3, a critical transcription factor, promotes the expression of downstream protective genes by recruiting methyltransferase complexes to enhance H3K4me3 levels [18]. WDR5, an essential component of the H3K4 methyltransferase complex, plays a central role in the SOX3-mediated epigenetic regulatory network [19,20]. Therefore, the construction of an engineered exosome system capable of targeting the transforming growth factor-β receptor (TGF-βR) and simultaneously activating the SOX3-H3K4me3 epigenetic axis and SIRT5-associated metabolic regulation may offer a promising dual-targeting strategy to mitigate chondrocyte death and metabolic dysregulation in OA.

The core objective of this study was to determine the therapeutic potential of a transforming growth factor-β receptor-targeted chondrocyte-derived exosome-nanoparticle complex (TGFβ-Exo-NP) in reversing OA progression. The study focused on the system's capacity to activate the SOX3-dependent H3K4me3 epigenetic modification axis and the SIRT5-mediated mitochondrial homeostasis pathway, thereby achieving coordinated regulation of apoptosis-, pyroptosis-, and ferroptosis-related changes and restoration of matrix metabolism. We proposed that the SOX3-H3K4me3-SIRT5 axis represents a key molecular cascade for OA intervention, with WDR5 serving as an exosomal cargo protein, acting as a regulatory hub within this signaling network. By elucidating this epigenetic-metabolic coupling mechanism, the study not only enriches the molecular understanding of OA pathogenesis but also establishes a novel paradigm for the targeted application of exosome-based nanosystems. This research holds significant scientific merit and translational potential, paving the way for breakthroughs in disease-modifying therapies and providing theoretical and practical foundations for precision medicine approaches to degenerative joint diseases.

## 2 Materials and methods

### 2.1 Experimental animals and ethical statement

All animal experiments were conducted in strict accordance with the Guide for the Care and Use of Laboratory Animals (8th Edition) and were approved by the Institutional Animal Care and Use Committee of Shanghai Changzheng Hospital. A nationally accredited animal experiment center provided specific pathogen-free (SPF) Sprague-Dawley (SD) rats. Animals were housed under standard conditions (temperature: 22 ± 2 °C; humidity: 55% ± 5%; 12-h light/dark cycle) with ad libitum access to food and water. All procedures were performed under sterile conditions, and efforts were made to minimize animal suffering. Postoperative analgesia was administered using buprenorphine (0.05 mg/kg). All operations were carried out by trained personnel to ensure compliance with ethical standards for animal welfare.

### 2.2 Primary chondrocyte isolation and OA model induction

To obtain chondrocytes with stable biological characteristics and a defined phenotype, articular cartilage was harvested under sterile conditions from the distal femur and proximal tibia of 4-week-old male SD rats (Beijing Vital River Laboratory Animal Technology, Catalog No.: 401). The cartilage was finely minced, pretreated with 0.25% trypsin for 15 min to remove surface proteins, and then digested in 0.2% collagenase II (Sigma-Aldrich, C6885) for 6 h at 37 °C in a 5% CO_2_ shaking incubator. Gentle pipetting was performed every 2 h to facilitate tissue dissociation. The resulting cell suspension was filtered through a 70 μm mesh, and the digestion was terminated by adding complete DMEM/F12 medium supplemented with 10% fetal bovine serum (FBS) and 1% penicillin-streptomycin. Cells were centrifuged, resuspended, and seeded into culture flasks. After cell adherence, nonadherent debris was removed, and the medium was replaced. Cells were cultured to 80-90% confluence and subsequently passaged using trypsinization.

For *in vitro* OA modeling, chondrocytes were seeded at a density of 2 × 10^5^ cells/well in 6-well plates. After 24 h, the medium was replaced with serum-free basal medium, followed by stimulation with recombinant human IL-1β (Peprotech, 10 ng/mL) for 24 h to induce an inflammatory OA phenotype [21]. Non-stimulated cells served as the negative control group and were cultured in parallel for all subsequent functional assays. In the intervention groups, TGFβ-Exo-NP or WDR5 knockdown (WDR5-KD) chondrocyte-derived exosome-nanoparticle complex (WDR5-KD-Exo-NP) was added.

### 2.3 Exosome isolation and purification

To prepare chondrocyte-derived exosomes, third-passage primary chondrocytes isolated from SD rats were used. After stable cell adherence was achieved, the cells were treated with recombinant human TGF-β1 (10 ng/mL, PeproTech) for 2 h. The stimulation medium was then discarded and replaced with DMEM/F12 supplemented with 1% exosome-depleted FBS (EV-depleted FBS), followed by continued culture for 48 h to collect the conditioned medium. EV-depleted FBS was prepared by subjecting conventional FBS to ultracentrifugation at 100,000 × g for 16 h at 4 °C to remove serum-derived exosomes, or alternatively, commercially available EV-depleted FBS (Gibco, A2720801) was used. During the culture period, cell morphology was monitored regularly to confirm the absence of obvious cell detachment or necrosis. After collection, the conditioned medium was processed by sequential differential centrifugation: centrifugation at 300 × g for 10 min to remove suspended cells, at 2000 × g for 20 min to eliminate cell debris, and at 10,000 × g for 30 min to exclude large extracellular vesicles and residual particles. The resulting supernatant was filtered through a 0.22 μm membrane and subjected to ultracentrifugation at 100,000 × g for 70 min at 4 °C to pellet the exosomes. After removal of the supernatant, the pellet was gently resuspended in sterile phosphate-buffered saline (PBS) and washed by a second round of ultracentrifugation at 100,000 × g for 70 min. The final exosome pellet was resuspended in PBS for subsequent experiments or characterization analyses.

### 2.4 Exosome characterization

The resulting exosome suspension was filtered through a 0.22 μm sterile membrane and resuspended in PBS for multidimensional characterization. Transmission electron microscopy (TEM, JEOL JEM-2100) was used to visualize the typical cup-shaped bilayer structure under an accelerating voltage of 80 kV. Nanoparticle tracking analysis (NTA, NanoSight NS300, Malvern) was performed to determine particle size distribution and concentration, with each sample recorded for 60 s and analyzed via automated trajectory fitting. Western blot (WB) was employed to detect exosome-specific markers, including CD63 (SBI, EXOAB-CD63A-1, 1:1000) and TSG101 (Abcam, ab125011, 1:1000). A total of 10 μg of protein was loaded per lane after quantification. Primary antibodies were incubated with PVDF membranes overnight at 4 °C, followed by incubation with an HRP-conjugated secondary antibody and visualization using enhanced chemiluminescence (ECL). The absence of GRP94 (Proteintech, 14700-1-AP, 1:2000) and Calnexin (Proteintech, 10427-2-AP, 1:2000) was confirmed, further validating the purity of the exosome preparation.

### 2.5 Flow cytometric detection of exosomal surface TGF-βRs

To verify the enrichment of TGF-βRs on the exosome surface, a magnetic bead-based exosome flow cytometry analysis was performed. Exosomes were first captured using anti-CD63 magnetic beads (Thermo Fisher Scientific, 10606D), allowing their immobilization on the bead surface for subsequent flow cytometric detection. The bead-bound exosomes were then incubated with primary antibodies, including anti-TGF-βR1 (Abcam, ab121024, 1:100) and anti-TGF-βR2 (Abcam, ab78419, 1:100), at 37 °C for 30 min in the dark, followed by thorough washing. FITC- or PE-conjugated secondary antibodies were subsequently applied for fluorescence detection. Corresponding isotype controls were used to correct for nonspecific binding. Fluorescence signals were acquired using a BD FACSAria III flow cytometer.

### 2.6 Construction of Exosome-LNP (lipid nanoparticle) functionalized nano-exosomes

Exosomes derived from TGF-β1-stimulated chondrocytes were used to construct a receptor-enriched exosome-LNP composite system. Purified exosomes were adjusted to a concentration of 1 × 10^11^ particles/mL and transferred into sterile low-protein-binding centrifuge tubes (Axygen, USA). The exosomes were obtained from third-passage chondrocytes isolated from SD rats following stimulation with recombinant human TGF-β1 (10 ng/mL, PeproTech, 100-21) for 2 h; the conditioned medium was filtered through a 0.22 μm membrane and purified by ultracentrifugation (Beckman Coulter Optima XPN-100, SW41Ti rotor, 100,000 × g for 70 min).

LNPs were prepared using the thin-film hydration-extrusion method and consisted of phospholipids, cholesterol, and PEG-modified lipids at predetermined molar ratios, yielding particles with diameters maintained within the range of 80-100 nm, as measured by a Malvern Zetasizer Nano ZS. Exosomes and LNPs were combined in HEPES buffer (pH 7.4, Thermo Fisher, 15630080) at an exosomal protein-to-total lipid mass ratio of 1:5, with a final volume of 200 μL. The mixture was incubated at 37 °C in a metal heating block (Eppendorf ThermoStat C) for 20 min with gentle agitation at 100 g to facilitate membrane fusion between the exosomal membrane and the LNP lipid bilayer.

After incubation, the mixture was centrifuged at 4000 × g for 15 min using 100 kDa ultrafiltration centrifugal filters (Amicon Ultra-0.5, Millipore, UFC510096) to remove unfused free LNPs and free exosomes, followed by two washes with PBS. The resulting exosome-LNP hybrid nano-exosomes were finally resuspended in 100 μL of calcium- and magnesium-free PBS for subsequent experiments.

### 2.7 Characterization of structural and physicochemical properties

The particle size distribution and zeta potential of the nanoparticles were measured using dynamic light scattering (DLS) on a Zetasizer Nano ZS (Malvern Instruments, UK). Samples were diluted in PBS to a final concentration of 0.5 mg/mL and measured in triplicate at 25 °C. Data were presented as mean ± standard deviation (mean ± SD). Morphological analysis was performed using TEM (JEOL JEM-2100, Japan). A drop of sample was placed on a Formvar/carbon-coated copper grid (Electron Microscopy Sciences) and negatively stained with 2% phosphotungstic acid for 30 s before observation at 80 kV. Particle size and morphology were quantified using ImageJ software based on at least 100 individual particles.

### 2.8 *In vitro* stability and release kinetics assay

To evaluate *in vitro* colloidal stability, TGFβ-Exo-NP suspensions were incubated in PBS or 10% FBS at 37 °C for 48 h, and hydrodynamic diameter was measured by DLS at 8, 16, 24, 32, 40, and 48 h. For release kinetics, TGFβ-Exo-NP suspensions were placed in dialysis membranes with a molecular weight cutoff of 12,000–14,000 Da and immersed in PBS at 37 °C with gentle shaking. At 0, 4, 8, 12, 16, 20, and 24 h, aliquots were collected for quantification, and the cumulative release percentage was calculated relative to the total loaded amount. Experiments were performed in triplicate.

### 2.9 Determination of exosome loading efficiency (Bicinchoninic acid [BCA] assay)

To quantify the loading efficiency of TGFβ-Exo onto poly(lactic-co-glycolic acid) (PLGA)/LNP nanoparticles, a BCA protein assay was used to calculate the recovery rate of exosomal protein in the TGFβ-Exo-NP complex. TGFβ-Exo suspension served as the input source, and a portion was reserved before membrane fusion to determine the initial total protein content. The exosome sample was lysed in RIPA buffer (Beyotime, P0013B) supplemented with 1× protease inhibitor (Roche, 04693159001) on ice for 30 min, with gentle pipetting every 10 min. Lysates were centrifuged at 12,000 × g for 15 min at 4 °C, and the supernatant was collected for protein quantification. A BCA Protein Assay Kit (Thermo Fisher, 23225) was used according to the manufacturer's instructions. Standard and test samples were added to a 96-well plate, incubated at room temperature for 30 min, and absorbance was measured at 562 nm using a Thermo Fisher Varioskan LUX microplate reader. Protein concentration was calculated based on a bovine serum albumin (BSA) standard curve and converted into total protein content, recorded as Protein_input_. Membrane fusion between TGFβ-Exo and PLGA/LNP was performed at a predetermined mass ratio. After fusion, the sample was purified using a 100 kDa ultrafiltration unit (Amicon Ultra-0.5, Millipore, UFC510096) by centrifugation at 4000 × g, 4 °C. The resulting TGFβ-Exo-NP fraction was collected, lysed using the same RIPA protocol, and quantified via BCA assay to determine the total protein content, recorded as Protein_bound_. The exosome loading efficiency was calculated using the following formula:

$$\text{Exosome}\;\text{loading}\;\text{efficiency}\;\left(\%\right)=\left({\text{Protein}}_{\text{bound}}/{\text{Protein}}_{\text{input}}\right)\times 100\%$$

### 2.10 Construction and validation of WDR5-KD-Exo-NP

To obtain donor cells capable of continuously secreting WDR5-KD-Exo, a stable WDR5-silenced chondrocyte line was first established. Third-passage chondrocytes were seeded in 6-well plates, and when cell confluence reached approximately 40-50%, lentiviral particles carrying WDR5-shRNA (OBiO, Shanghai; viral titer ≥1 × 10^8^ TU/mL) were added at a multiplicity of infection (MOI) of 10. Polybrene (8 μg/mL, Sigma, H9268) was included to enhance transduction efficiency. After 24 h of infection, the medium was replaced with fresh complete culture medium and incubation continued for another 48 h. Puromycin (2 μg/mL, InvivoGen, ant-pr-1) was then added for antibiotic selection, which lasted 5-7 days until all uninfected control cells had died. The surviving stable clones were cultured for an additional 72 h and assessed by WB (WDR5 antibody: Cell Signaling Technology [CST], 13105) to confirm sustained WDR5-KD protein expression.

Once the WDR5-KD chondrocytes reached 80% confluency, the medium was replaced with EV-depleted medium containing 2% FBS (Gibco, A2720801), and the cells were cultured for an additional 48 h to collect conditioned supernatants. Exosomes were isolated using differential ultracentrifugation: sequential centrifugation at 300 × g for 10 min to remove cell debris, 2000 × g for 20 min to eliminate large particles, 10,000 × g for 30 min to exclude microvesicles, and finally 100,000 × g for 70 min (Beckman Optima XPN-100, SW41Ti rotor) to pellet exosomes. The pellet was gently resuspended in PBS and washed again by ultracentrifugation at 100,000 × g for 70 min. The final exosome pellet was resuspended in 100 μL PBS and quantified using the BCA protein assay (Thermo Fisher, 23225), yielding WDR5-KD-Exo. These exosomes were then loaded into PEG-PLGA nanoparticles at a concentration of 50 μg/mL (see *Construction of Functionalized Exosomes via Exosome- PLGA/LNP Membrane Fusion* section for details), and the mixture was incubated at 37 °C for 20 min to facilitate membrane fusion.

To confirm the localization of WDR5 within the exosomes, a proteinase K protection assay was performed. Exosome samples were divided into three groups: (1) PBS control; (2) proteinase K (Thermo, EO0491, 50 μg/mL) treatment; (3) combined treatment with proteinase K and Triton X-100. After 30 min of incubation at 37 °C, the protease inhibitor was immediately added to terminate the reaction.

#### 2.10.1 FITC labeling and cellular uptake assay

To visualize the cellular uptake of TGFβ-Exo-NP, exosomes were fluorescently labeled with FITC (Thermo Fisher Scientific, 46410). Exosome samples (100 μg total protein) were dissolved in 1 mL PBS and incubated with FITC solution (1 mg/mL) at a final concentration of 100 μg/mL. The mixture was gently shaken in the dark at 4 °C for 2 h. Unbound FITC molecules were removed by three rounds of centrifugation using Amicon Ultra-100K filters (Millipore, UFC910024) at 4000 × g for 10 min each. Labeling efficiency was confirmed by measuring fluorescence intensity at an excitation wavelength of 488 nm and emission at 520 nm using a fluorescence spectrophotometer (Thermo Varioskan LUX). Chondrocytes were seeded onto glass chamber slides (Nunc Lab-Tek, Thermo Fisher), and once cell confluence reached 70-80%, FITC-labeled TGFβ-Exo-NPs were added to the culture medium (DMEM/F12 with 10% exosome-depleted FBS, Gibco, 11330-032) at a final concentration of 50 μg/mL. Cells were incubated for 0, 2, 4, 8, and 12 h, respectively. After incubation, cells were washed three times with PBS, fixed with 4% paraformaldehyde for 15 min, and permeabilized using 0.1% Triton X-100 (Sigma, T8787) for 10 min. Nuclei were counterstained with DAPI (Thermo Fisher, D1306, 1 μg/mL) for 5 min, and slides were mounted for imaging. Intracellular fluorescence distribution was observed using a Zeiss LSM880 confocal laser scanning microscope (Carl Zeiss, Germany) with a 63× oil immersion objective. Z-stack images were acquired at 0.3 μm intervals. Excitation/emission wavelengths were set to 488/520 nm for FITC and 358/461 nm for DAPI. For each group, at least five random fields were imaged to ensure statistical representativeness.

In addition, to evaluate the receptor-dependent cellular uptake of TGFβ-Exo-NP, cells were pretreated with the TGF-βR kinase inhibitor SB431542 (Sigma-Aldrich, S4317) at a final concentration of 10 μM for competitive blockade prior to exosome administration.

### 2.11 Subcellular Co-localization and lysosomal escape analysis

To evaluate the lysosomal escape efficiency of TGFβ-Exo-NP, lysosomes were labeled using LysoTracker™ Red DND-99 (Invitrogen, L7528). After co-incubation of cells with TGFβ-Exo-NP for the indicated time points, LysoTracker Red (50 nM) was added and incubated at 37 °C for 30 min. Cells were then washed three times with PBS, and images were immediately captured. Three fluorescence channels were used to separately record signals from DAPI, FITC, and LysoTracker, with proper settings to avoid spectral overlap. Image analysis was performed using the Coloc2 plugin in ImageJ/Fiji (NIH, USA) to calculate Pearson's correlation coefficient (*r* value), which quantified the degree of co-localization between exosome (FITC) and lysosomal signals. The Line Profile tool was used to extract fluorescence intensity curves, enabling direct visualization of the spatial distribution and dissociation trend between FITC and LysoTracker signals. Additionally, the proportion of FITC-positive cells and their mean fluorescence intensity (MFI) were measured using a flow cytometer (BD LSRFortessa, BD Biosciences, USA).

### 2.12 JC-1 staining

JC-1 staining (Beyotime, C2006) was used to assess changes in mitochondrial membrane potential. Following treatment, cells were incubated with 5 μg/mL JC-1 dye at 37 °C for 30 min. After washing with PBS, fluorescence images were immediately acquired using a confocal laser scanning microscope. JC-1 aggregates emitted red fluorescence in cells with high membrane potential, while monomers emitted green fluorescence under depolarized conditions. The red-to-green fluorescence intensity ratio was calculated in ImageJ.

### 2.13 MitoTracker red staining

Chondrocytes were seeded in 24-well plates containing glass coverslips. Following the designated treatments, the culture medium was removed, and cells were rinsed with pre-warmed serum-free DMEM/F12. MitoTracker™ Red CMXRos (Invitrogen, M7512) was diluted to a final concentration of 100 nM and added to the cells for incubation at 37 °C in 5% CO_2_ for 30 min in the dark. After incubation, cells were washed twice with PBS, fixed with 4% paraformaldehyde for 15 min, and washed three additional times with PBS. If required, nuclei were counterstained with DAPI (1 μg/mL) for 5 min. Images were acquired using a confocal microscope with excitation at 561 nm, and identical imaging parameters were applied across all groups. Fluorescence intensity and mitochondrial morphology were analyzed using ImageJ/Fiji software. Background fluorescence was subtracted before calculating average intensity, which was subsequently normalized. Each group included at least three biological replicates.

### 2.14 Intracellular reactive oxygen species (ROS) level detection

After chondrocytes underwent the assigned treatments, the culture medium was removed, and cells were gently rinsed once with pre-warmed PBS. According to the manufacturer's instructions, the DCFH-DA probe (Beyotime, S0033S) was diluted in serum-free medium to a final concentration of 10 μM and added to each well to fully cover the cells. Cells were incubated for 30 min at 37 °C under 5% CO_2_ in the dark. After incubation, cells were washed three times with PBS to remove excess probe, and green fluorescence images were immediately captured using an inverted fluorescence microscope (Ex/Em ≈ 488/525 nm). Exposure settings were kept consistent across all groups. For each well, at least five random fields were imaged. The average fluorescence intensity per cell was calculated using ImageJ and normalized to the control group. Results were expressed as relative ROS levels.

### 2.15 ATP quantification by colorimetric assay

Following treatment, cells were washed once with cold PBS and lysed using the lysis buffer provided in the ATP Colorimetric Assay Kit (Beyotime, S0026). Lysis was performed on ice for 30 min, followed by centrifugation at 12,000 × g for 5 min at 4 °C. The supernatant was collected for analysis. A standard curve was prepared according to the kit instructions. Cell lysates were mixed with the ATP colorimetric working solution and incubated at 37 °C for 30 min. Absorbance was measured at 570 nm using a microplate reader. ATP levels were calculated based on the standard curve and normalized to total protein concentration as determined by the BCA assay. Results were expressed as relative ATP levels, with at least three independent biological replicates per group.

### 2.16 Raman spectroscopy for energy metabolism assessment

To investigate subtle changes in energy metabolism, Raman spectra were acquired under label-free conditions using a DXR Raman microscope (Thermo Scientific). Spectral features related to ATP synthesis, NAD^+^/NADH redox states, and membrane phospholipid organization were specifically monitored.

### 2.17 Annexin V-FITC/propidium iodide (PI) flow cytometry

To evaluate the effect of exosome intervention on apoptosis in OA chondrocytes, Annexin V-FITC/PI dual staining was used to detect both early and late apoptosis via flow cytometry. After 24 h of treatment, chondrocytes were gently digested with trypsin (without EDTA, Gibco, 25200-072), washed twice with PBS (pH 7.4), and resuspended in Annexin V binding buffer (BD Pharmingen, 556454). Cells were stained with 5 μL each of Annexin V-FITC and PI (BD Pharmingen, 556547), incubated in the dark for 15 min, and analyzed immediately. Data were acquired using a BD FACSCalibur flow cytometer (BD Biosciences, USA). All experiments were performed in triplicate under temperature-controlled (25 °C) and light-protected conditions to ensure dye stability.

### 2.18 Alcian Blue staining

To functionally evaluate the effects of TGFβ-Exo-NP on the matrix synthetic capacity of chondrocytes, Alcian Blue staining was performed to assess glycosaminoglycan (GAG) deposition under different treatment conditions. Chondrocytes were seeded into six-well plates at a density of 2 × 10^5^ cells per well and cultured in DMEM/F12 medium supplemented with 10% FBS for 24 h to allow cell attachment. The medium was then replaced with serum-free medium, and recombinant human IL-1β (10 ng/mL, PeproTech, Cat# 200-01B) was added for 24 h to establish an *in vitro* inflammatory chondrocyte injury model; subsequently, cells were treated with TGFβ-Exo-NP and cultured for an additional 48 h. After treatment, cells were washed twice with PBS and fixed with 4% paraformaldehyde for 30 min. The fixed cells were pretreated with 0.1 mol/L hydrochloric acid for 10 min to enhance staining specificity, followed by incubation with 1% Alcian Blue 8GX staining solution (pH 2.5, Sigma-Aldrich, Cat# A5268) at room temperature in the dark for 30 min. After staining, cells were thoroughly rinsed with deionized water to remove nonspecifically bound dye, and representative images were captured using an inverted optical microscope (Olympus IX73, Olympus Corporation, Japan). Quantitative analysis was performed using ImageJ software (National Institutes of Health, Version 1.53).

### 2.19 WB

Treated cells were lysed in RIPA buffer (Beyotime, P0013B) supplemented with protease and phosphatase inhibitors (Roche, 04693159001), and incubated on ice for 30 min. Lysates were centrifuged at 12,000 × g for 10 min at 4 °C, and the supernatant was collected. Protein concentrations were determined using a BCA Protein Assay Kit (Thermo Scientific, 23225). Histones were extracted using acid extraction. Briefly, cells were washed with PBS and resuspended in 0.2 M H_2_SO_4_, then rotated for 1 h to release histones. The suspension was then centrifuged at 12,000 × g for 10 min at 4 °C. The supernatant was precipitated with 20% trichloroacetic acid (Sigma, T6399) to a final concentration of 10%, incubated on ice for 1 h, and centrifuged again at 4 °C. Pellets were washed twice with acetone, air-dried, and resuspended in ddH_2_O. After quantification, the extracts were used to detect H3K4me3 and total H3. For WB, 30 μg of protein per lane was separated by 10-12% SDS-PAGE using the Mini-PROTEAN Tetra System (Bio-Rad), and transferred onto PVDF membranes (Millipore, IPVH00010). Membranes were blocked in 5% skim milk (BD Difco) for 1 h at room temperature. Primary antibodies were incubated overnight at 4 °C and included the following: anti-SOX3 (Abcam, ab183606, 1:1000), anti-PRMT5 (Abcam, ab109451, 1:1000), anti-SIRT5 (CST, #8782, 1:1000), anti-Aggrecan/ACAN (Proteintech, 13880-1-AP, 1:1000), anti-cleaved caspase-3 (CST, #9661, 1:1000), anti-IL-6 (CST, #12912, 1:1000), anti-MMP13 (Abcam, ab39012, 1:1000), anti-SOX9 (CST, #82630, 1:1000), anti-COL2A1 (Abcam, ab34712, 1:1000), anti-H3K4me3 (CST, #9751, 1:1000), anti-GSDMD-N (Abcam, ab215203, 1:1000), anti-cleaved caspase-1 (p20) (CST, #4199, 1:1000), anti-FTH1 (CST, #3998, 1:1000), anti-GPX4 (Abcam, ab125066, 1:1000), anti-ACSL4 (Abcam, ab155282, 1:1000), anti-SLC7A11 (Proteintech, 32384-1-AP, 1:1000), anti-NLRP3 (AdipoGen, AG-20B-0014, 1:1000), anti-ASC (Santa Cruz, sc-514414, 1:500), β-actin (CST, #4970, 1:5000), or anti-total H3 (CST, #4499, 1:2000) as the loading control. HRP-conjugated secondary antibodies against rabbit or mouse IgG (Jackson ImmunoResearch, 111-035-003/115-035-003, 1:5000) were incubated at room temperature for 1 h. Protein bands were visualized using ECL (Thermo, 32106) and captured with the ChemiDoc MP imaging system (Bio-Rad, USA). Densitometric analysis was performed using ImageJ software (NIH, USA). Band intensities were normalized to internal controls and presented as relative expression levels (fold change).

### 2.20 Reverse transcription quantitative polymerase chain reaction (RT-qPCR)

Total RNA was extracted using TRIzol™ Reagent (Invitrogen, 15596026, USA) according to the manufacturer's instructions. RNA purity was assessed by measuring the OD_260/280_ ratio using a NanoDrop 2000 spectrophotometer (Thermo Fisher Scientific, USA), and samples with ratios between 1.8 and 2.0 were used for downstream analysis. RNA integrity was verified by 1% agarose gel electrophoresis to exclude degradation. For each group, 1 μg of total RNA was reverse transcribed using the PrimeScript™ RT Reagent Kit with gDNA Eraser (Takara, RR047A, Japan) on a GeneAmp PCR System 9700 thermal cycler (Applied Biosystems, USA). The 20 μL reaction volume was incubated at 42 °C for 2 min (genomic DNA removal), followed by reverse transcription at 37 °C for 15 min, and terminated at 85 °C for 5 s. Quantitative polymerase chain reaction (qPCR) was performed using SYBR® Premix Ex Taq™ II (Takara, RR820A, Japan) on a QuantStudio™ 6 Flex Real-Time PCR System (Applied Biosystems, USA). The 20 μL reaction mixture consisted of 10 μL SYBR Premix, 0.4 μL ROX Reference Dye, 0.4 μL each of forward and reverse primers (10 μM), 2 μL cDNA template, and DEPC-treated nuclease-free water to adjust the volume. The amplification protocol was as follows: initial denaturation at 95 °C for 30 s, followed by 40 cycles of 95 °C for 5 s and 60 °C for 30 s. Each sample was run in technical triplicate. Melting curve analysis was performed to assess amplification specificity, with a single-peak profile considered acceptable. Target genes included SOX3, IL-6, MMP13, Sox9, and Col2a1. The reference gene was β-actin. Primers were synthesized by Sangon Biotech (Shanghai, China) and optimized by gradient PCR to ensure amplification efficiency between 90% and 110%. The primer sequences were as follows:

SOX3: F: 5′-TATAGCCAGGTGTGCGTGTG-3′, R: 5′-ACCCGAACGAAATGCGTACA-3′

IL-6: F: 5′-CCACCCACAACAGACCAGTA-3′, R: 5′-GTCTTGGTCCTTAGCCACTCC-3′

MMP13: F: 5′-TGCTGCATACGAGCATCCAT-3′, R: 5′-TGTCCTCAAAGTGAACCGCA-3′

Sox9: F: 5′-TCCCCGCAACAGATCTCCTA-3′, R: 5′-AGCTGTGTGTAGACGGGTTG-3′

Col2a1: F: 5′-TCCTACAATGTCAGGGCCAG-3′, R: 5′-GTTGAGGCAGTCTGGGTCTT-3′

β-actin: F: 5′-AGGGAAATCGTGCGTGACAT-3′, R: 5′-GCAGCTCAGTAACAGTCCGC-3′

All Ct values were automatically generated by QuantStudio Real-Time PCR Software (v1.3, Applied Biosystems). Relative gene expression levels were calculated using the 2^−ΔΔCt^ method: ΔCt = Ct_target_ - Ct_reference_, ΔΔCt = ΔCt_treated_ - ΔCt_control_.

### 2.21 Chromatin immunoprecipitation followed by quantitative polymerase chain reaction (ChIP-qPCR)

To further investigate the role of H3K4me3 modification in the epigenetic activation of SOX3 target genes (PRMT5 and SIRT5), ChIP-qPCR was performed. The assay was conducted using a ChIP Assay Kit (Millipore, 17-295, USA) with protocol optimization. Cells were cross-linked with 1% formaldehyde (Sigma, F8775) at room temperature for 10 min, and the reaction was quenched with 125 mM glycine. After lysis, chromatin was sonicated using a Bioruptor Plus (Diagenode, Belgium) with a cycle of 30 s on/30 s off for 15 min to generate DNA fragments of 200-500 bp. Debris was removed by centrifugation, and the supernatant was used for immunoprecipitation.

The immunoprecipitation mixture contained anti-H3K4me3 antibody (CST, #9751, 1:100) and Protein A/G magnetic beads (Thermo, 10002D), and was incubated overnight at 4 °C with rotation. Rabbit IgG (Millipore, 12-370) served as the negative control. The complexes were washed sequentially with low-salt, high-salt, LiCl, and TE buffers. Cross-links were reversed by incubation at 65 °C for 4 h. The samples were then treated with RNase A (Thermo, EN0531) and Proteinase K (Thermo, EO0491), followed by DNA purification using the QIAquick PCR Purification Kit (Qiagen, 28104, Germany), and elution in EB buffer. Purified DNA was subjected to qPCR using SYBR Green Master Mix (Takara, RR820A, Japan) on the QuantStudio™ 6 Flex Real-Time PCR System (Applied Biosystems, USA). Primers were designed to target H3K4me3-enriched regions within the promoter regions of SOX3, PRMT5, and SIRT5:

SOX3-promoter: F: 5′-TCCCTGTGCTCTGGGTTTAG-3′, R: 5′-GGGACTCTGGCTTCTGTTTG-3′

PRMT5-promoter: F: 5′-CGTCTGTGGCCTCTTACCTC-3′, R: 5′-TCTGCTCAGGTGATCCTTGC-3′

SIRT5-promoter: F: 5′-CTCAGGTCGAGGAGTTTGGA-3′, R: 5′-CGGTTTGGGACGACTGTTTA-3′

Relative enrichment was calculated using the input normalization method:

$$\text{Fold}\;\text{Enrichment}=2^{‐\left[C_{t}\left(\text{IP}\right)‐C_{t}\left(\text{Input}\right)\right]}$$

### 2.22 Immunofluorescence double staining and confocal imaging analysis

To verify the spatial interaction between SOX3 and H3K4me3 within the nucleus following TGFβ-Exo-NP treatment, immunofluorescence double staining combined with confocal microscopy was employed. Chondrocytes were seeded onto 24-well chambered coverslips (Thermo Fisher, 12565380) and cultured until reaching 60-70% confluence. After 24 h of treatment under various conditions, cells were fixed with 4% paraformaldehyde (Solarbio, P1110), permeabilized with 0.3% Triton X-100 (Sigma, T8787), and blocked with 5% BSA (Sigma, A9647). Cells were then incubated overnight at 4 °C with primary antibodies against SOX3 (Abcam, ab183606, 1:200) and H3K4me3 (CST, #9751, 1:400). On the following day, cells were incubated in the dark for 1 h with Alexa Fluor 488-conjugated anti-rabbit IgG (Invitrogen, A-11008, 1:500) and Alexa Fluor 594-conjugated anti-mouse IgG (Invitrogen, A-11012, 1:500). Nuclei were counterstained with DAPI (Thermo, D1306, 1 μg/mL), and coverslips were mounted using an antifade reagent (Vector, H-1000). Imaging was performed using a Zeiss LSM880 confocal laser scanning microscope (Carl Zeiss, Germany).

### 2.23 Establishment of the SD rat destabilization of the medial meniscus (DMM) model and group interventions

Male specific pathogen-free SD rats aged 6 weeks and weighing 220-250 g were obtained from Beijing Vital River Laboratory Animal Technology (Catalog No.: 401). Under isoflurane inhalation anesthesia (maintenance concentration, 2.5%), DMM surgery was performed on the right knee joint. After routine shaving and disinfection of the surgical area, a longitudinal incision was made along the medial aspect of the knee, and the soft tissues were bluntly dissected layer by layer to expose the medial joint capsule, which was then incised to access the joint cavity. Under direct visualization, the anterior horn region of the medial meniscus was manipulated, and the ligamentous attachment between the medial meniscus and the tibial plateau was transected. After confirming loosening of the medial meniscus, the joint cavity was irrigated with sterile saline, followed by layered closure of the joint capsule and skin incision. Throughout the procedure, the anterior cruciate ligament (ACL) was preserved intact and was not transected.

On postoperative day 7, intervention treatments were initiated. Using a random number table, rats were equally divided into five groups (n = 10 per group): Sham, DMM model, TGF-β-targeting nanoparticle group (TGFβ-Exo-NP), SOX3 knockdown (shSOX3) group (TGFβ-Exo-NP + shSOX3), and the WDR5-KD-Exo-NP group. Intra-articular injections began on day 7 post-surgery, with each administration consisting of 50 μL of formulation per knee. The working concentration of TGFβ-Exo-NP or WDR5-KD-Exo-NP was 1 × 10^10^ particles/mL. Injections were performed once weekly for four consecutive weeks [22]. In the TGFβ-Exo-NP + shSOX3 group, AAV-shSOX3 was co-administered during the first and third nanoparticle injections. The recombinant adeno-associated virus used was AAV-shSOX3 (serotype AAV9, titer 1 × 10^13^ vg/mL, OBiO Technology, China). The viral sequence was validated for effective targeting by double-enzyme digestion and Sanger sequencing. Before administration, the virus suspension was thawed on ice and gently mixed. A total of 10 μL (1 × 10^11^ vg per knee) was injected intra-articularly using a microsyringe fitted with a 33G needle. Under mild isoflurane anesthesia, rats were positioned with the knee flexed, and the needle was inserted adjacent to the patellar ligament. Once intra-articular access was confirmed, the solution was slowly injected over 20-30 s, and the needle was held in place for an additional 5 s to prevent reflux. Throughout the procedure, sterile technique was maintained. After injection, animals were monitored for mobility, with no signs of puncture injury or leakage observed. Stable shSOX3 was achieved within 7-10 days after AAV administration and remained effective throughout the subsequent TGFβ-Exo-NP dosing period.

### 2.24 Toxicological analysis

To evaluate the systemic biosafety of TGFβ-Exo-NP, the TGFβ-Exo-NP working solution was prepared at a concentration of 1 × 10^10^ particles/mL and administered intra-articularly once weekly for 4 consecutive weeks at a dose of 50 μL per knee. After the intervention, orbital blood was collected from mice for serum biochemical analysis. Alanine aminotransferase (ALT), aspartate aminotransferase (AST), blood urea nitrogen (BUN), and creatinine (Cr) levels were measured using a Hitachi 7600 automatic biochemical analyzer to assess liver and kidney function.

Major organs, including the heart, liver, lung, and kidney, were collected simultaneously, fixed in 4% paraformaldehyde, dehydrated, embedded in paraffin, sectioned at 5 μm, and subjected to hematoxylin and eosin (H&E) staining. Tissue architecture, cellular degeneration, edema, inflammatory infiltration, and necrotic changes were examined microscopically to evaluate whether TGFβ-Exo-NP induced organ toxicity and systemic inflammatory responses. These analyses were used to establish systemic safety evidence supporting the molecular specificity and chondroprotective effects of TGFβ-Exo-NP.

### 2.25 Gait behavior assessment and quantitative analysis

On postoperative day 28, the gait behavioral characteristics of rats in each experimental group were systematically evaluated. To minimize the influence of environmental stress on gait performance, all rats underwent adaptive training in the gait corridor for three consecutive days prior to testing. Gait assessment was performed using a standardized footprint analysis method: the forepaws and hind paws were separately marked with non-toxic, water-based paint, after which the rats were guided to traverse a closed gait corridor (50 cm in length and 8 cm in width) under voluntary walking conditions. High-resolution white recording paper was placed along the corridor floor to capture continuous and clearly defined footprint patterns. For each rat, at least three complete, uninterrupted walking trials were collected for subsequent analysis. Gait parameters, including mean stride width, stride length, and toe spread, were quantified, with all measurements derived from hind paw footprints.

### 2.26 Forelimb grip strength test

Forelimb muscle strength was evaluated using a grip strength meter (Columbus Instruments, USA; Model 1027SM). During testing, the rat's tail was gently held to encourage natural gripping of a metal mesh bar with its forepaws. The animal was then pulled back horizontally at a constant speed until its grip was released, and the peak tension (in Newtons) was automatically recorded by the system. Each rat underwent three consecutive trials, with a 30-s rest between trials to avoid muscle fatigue. The average of the three measurements was taken as the final result for each individual. All tests were conducted by the same trained operator to reduce inter-operator variability and were performed between 9:00 and 11:00 a.m. to control for circadian influences.

### 2.27 Knee circumference measurement and swelling assessment

Knee joint circumference was measured using a non-elastic nylon tape (Boley, Germany; accuracy: 0.1 mm) horizontally wrapped around the lower margin of the patella. Measurements were conducted preoperatively (baseline) and on postoperative days 14 and 28. Each measurement was repeated three times, and the mean value was calculated. The change in circumference relative to baseline (Δ circumference) was used to evaluate joint swelling and effusion. During measurement, animals were maintained under light isoflurane anesthesia, with the knee extended naturally at an angle of 120° ± 5°. All measurements were performed by two independent investigators using a double-blinded protocol, with separate operators for measurement and recording. Results were independently verified, and intergroup differences were calculated.

### 2.28 Micro-computed tomography (Micro-CT) imaging and bone microarchitecture quantification

Micro-CT scans were performed using the Skyscan 1176 system (Bruker, Belgium) under the following conditions: 70 kV voltage, 200 μA current, 0.5 mm aluminum filter, and spatial resolution of 9-12 μm/voxel. After image reconstruction using NRecon software, trabecular bone parameters including bone volume fraction (BV/TV), trabecular thickness (Tb.Th), and trabecular number (Tb.N) were quantified within a standardized region of interest (ROI) using CTAn software (Bruker). A calibration phantom was used daily to ensure accuracy, and metal artifacts were minimized through optimized thresholding. Three-dimensional visualizations were rendered using CTvol software.

### 2.29 Tissue harvesting and histological processing

Immediately after euthanasia, the knee joints were flushed with saline to remove residual blood. Based on anatomical landmarks, the femoral distal and tibial proximal segments were harvested as an intact unit. Samples were fixed in 4% paraformaldehyde (Solarbio, P1110) at room temperature for 24 h, followed by decalcification in 10% EDTA solution (pH 7.4; Solarbio, E1171) at 4 °C with gentle agitation on a magnetic stirrer for 2-3 weeks. The decalcification solution was replaced every 48 h, and endpoint determination was verified by needle probing and X-ray imaging. Tissues were then dehydrated through a graded ethanol series (70%-100%), cleared with analytical-grade xylene (China National Pharmaceutical Group), and embedded in paraffin using an automated embedding system (Leica EG1150). Orientation was adjusted to ensure coronal sections traversed both medial and lateral tibial plateaus. Serial sections were cut at 5 μm thickness using a rotary microtome (Leica RM2235), mounted on positively charged glass slides (Thermo Superfrost Plus, J1800AMNZ), and baked at 60 °C for 1 h before staining.

### 2.30 Hematoxylin and eosin (H&E) and safranin O/Fast Green staining and OARSI scoring

Following deparaffinization and rehydration, H&E and Safranin O/Fast Green staining were performed according to the manufacturer's instructions (Servicebio, China; G1371/G1034). Hematoxylin counterstaining was limited to 30-60 s to avoid overstaining. After dehydration in graded ethanol and clearing in xylene, sections were mounted in neutral balsam (Solarbio, G8590). For each knee joint, 3-5 coronal sections spaced 100 μm apart were selected for scoring. OA severity was assessed using the OARSI scoring system (Grade 0-4 × Stage 0-6) by two trained pathologists blinded to group allocation. When score differences exceeded 1 point, a third expert adjudicated. Group means and inter-rater reliability were reported. Whole-slide images were acquired using a digital slide scanner (3DHISTECH Pannoramic or Leica Aperio) at 20× magnification, and exported as 16-bit TIFF files for further analysis.

### 2.31 Immunohistochemistry (IHC)

After deparaffinization and rehydration, endogenous peroxidase activity was blocked by incubating the sections in 3% hydrogen peroxide at room temperature for 10 min. Antigen retrieval was performed using either citrate buffer (pH 6.0; ZSGB-BIO, ZLI-9064) or EDTA buffer (pH 9.0; ZSGB-BIO, ZLI-9065), followed by microwave heating at 95-100 °C for 10-15 min and natural cooling. Non-specific binding was blocked with 5% goat serum (Beyotime, C0265) for 30 min at room temperature. Primary antibodies were incubated overnight at 4 °C in a humidified chamber: anti-SOX3 (Abcam, ab183606, 1:200), anti-H3K4me3 (CST, #9751, 1:400), anti-COL2A1 (Abcam, ab34712, 1:200), anti-SOX9 (CST, #82630, 1:200), anti-MMP13 (Abcam, ab39012, 1:200), and anti-cleaved caspase-3 (CST, #9661, 1:300). On the following day, sections were washed with PBS and incubated with a polymer-based HRP detection system (DAKO EnVision+, K5007) at room temperature for 30 min. Visualization was achieved using DAB substrate (DAKO, K3468), followed by hematoxylin counterstaining, dehydration, and mounting. Negative controls included isotype-matched IgG and no-primary antibody controls; known positive tissues were included as internal references. Antibody batch numbers and dilutions were recorded. To minimize batch-to-batch variability, all targets were processed in parallel using identical staining protocols, and endpoint detection was optimized using test slides with graded chromogen exposure. For IHC quantification, the Color Deconvolution plugin in ImageJ/Fiji (Ruifrok's method, DAB-Hematoxylin vector) was applied to extract the DAB channel. Otsu or adaptive thresholding was used to segment positive staining areas, and both the percentage of positive area and the integrated optical density (IOD/Area) were calculated.

### 2.32 RNA sequencing (RNA-seq) and analysis

RNA-seq was performed on primary chondrocytes subjected to two treatment conditions: IL-1β and IL-1β combined with TGFβ-Exo-NP. Each group included three independent biological replicates. Total RNA was extracted using TRIzol (Invitrogen), followed by rRNA depletion or poly(A) enrichment and library construction with the NEBNext Ultra II kit (NEB, E7770). Paired-end sequencing (PE150) was conducted on the Illumina NovaSeq 6000 platform (Illumina). After quality control (QC) with fastp, reads were aligned to the Rnor_6.0 reference genome using STAR (v2.7), and gene-level quantification was performed with featureCounts. Differential expression analysis was conducted using DESeq2 (R v4.3), with thresholds set at |log_2_FC| ≥ 1 and *p-*value <0.05. Gene Ontology (GO) and Kyoto Encyclopedia of Genes and Genomes (KEGG) pathway enrichment analyses were performed using clusterProfiler (GO: http://geneontology.org/; KEGG: https://www.kegg.jp/), and the results were visualized with EnhancedVolcano and ggplot2. Both raw and processed data were submitted to the NCBI GEO database.

### 2.33 Chromatin immunoprecipitation sequencing (ChIP-seq; H3K4me3)

ChIP-seq analysis was performed on chondrocytes treated with IL-1β or IL-1β + TGFβ-Exo-NP, with three independent biological replicates per group. Chromatin immunoprecipitation was performed using a specific anti-H3K4me3 antibody (Cell Signaling, #9751), and all experimental steps—including cross-linking, chromatin shearing, immunoprecipitation, and DNA purification—were carried out using the ChIP-IT High Sensitivity Kit. Libraries were constructed and sequenced, and reads were aligned to the reference genome using Bowtie2. Peak calling was performed using MACS2 to identify significant enrichment regions. Peak annotation was completed with the ChIPseeker package, focusing on those localized to promoter regions. Visualization and alignment of ChIP peaks were performed using IGV, allowing for identification of candidate genes regulated by H3K4me3, with particular emphasis on signaling pathways co-regulated with SOX3.

### 2.34 TMT-based quantitative proteomics

TMT-based quantitative proteomic analysis was performed on chondrocytes under two treatment conditions: IL-1β and IL-1β + TGFβ-Exo-NP. Each group included five independent biological replicates. Proteins were extracted, digested with trypsin, and labeled using the TMTpro 16-plex kit (Thermo, A44520). Nano-scale liquid chromatography-tandem mass spectrometry (LC-MS/MS) was conducted on the Orbitrap Exploris 480 platform (Thermo). Database searching was carried out with MaxQuant (v2.0) against the UniProt databases for Rattus, Mus, and Homo, with a false discovery rate (FDR) controlled at 1%. Differentially expressed proteins (DEPs) were identified using a threshold of |log_2_FC| ≥ 1 and *p*-value <0.05. Functional enrichment analyses were performed based on GO and KEGG pathway annotations.

### 2.35 Untargeted metabolomics

To determine whether SOX3-mediated H3K4me3 epigenetic regulation induces metabolic reprogramming in OA chondrocytes, untargeted metabolomic profiling was conducted following exosome intervention. Two treatment groups were analyzed: IL-1β and IL-1β combined with TGFβ-Exo-NP, each with six independent biological replicates. Cell culture supernatants were collected and stored at −80 °C. Before analysis, proteins were precipitated using a pre-chilled methanol:acetonitrile mixture (1:1, v/v). After centrifugation at 10,000 × g for 15 min at 4 °C, the supernatants were collected for LC-MS analysis. Metabolomic profiling was performed using the Thermo Q Exactive Plus mass spectrometer equipped with an HSS T3 C18 column (2.1 × 100 mm, 1.8 μm). The mobile phase consisted of water (0.1% formic acid) and acetonitrile (0.1% formic acid). Data acquisition was performed in both positive and negative ionization modes, covering an *m*/*z* range of 70-1000.

Raw data were processed using Compound Discoverer 3.1 for peak detection, alignment, and normalization. QC samples were used to monitor instrument stability throughout the run. Metabolite identification was based on matching with the Human Metabolome Database (HMDB) and mzCloud, using variable importance in projection (VIP) > 1 and *p < *0.05 as filtering criteria. Differential metabolites were further analyzed using MetaboAnalyst 5.0 for principal component analysis (PCA) and partial least squares discriminant analysis (PLS-DA) to evaluate intergroup metabolic divergence. Pathway enrichment was conducted using the KEGG database (https://www.genome.jp/kegg/).

In addition, key indicators of energy metabolism were quantitatively analyzed across treatment groups to validate the epigenetic axis's direct regulatory role in cellular metabolic flux.

### 2.36 Exosomal functional component analysis

To comprehensively characterize the bioactive molecular components of TGF-βR-enriched chondrocyte-derived exosomes, two treatment groups were established: IL-1β and IL-1β + TGFβ-Exo-NP, with five independent biological replicates per group. Total exosomal proteins were first isolated and quantified. Proteomic profiling was performed using a Thermo Scientific Orbitrap Elite LC-MS/MS platform. Following standardized lysis and trypsin digestion protocols, peptide mixtures were separated by high-performance liquid chromatography (EASY-nLC 1200) and analyzed on an Orbitrap mass spectrometer using full MS scans coupled with data-dependent MS/MS acquisition. Raw data were processed using Proteome Discoverer (v2.4), and protein identification was conducted by searching against the UniProt *Rattus norvegicus* database. Search parameters were set as follows: precursor mass tolerance <10 ppm, fragment mass tolerance <0.02 Da, and identification of at least one unique peptide per protein. A FDR <1% was applied for protein filtering. Identified proteins were subsequently subjected to GO annotation, KEGG pathway enrichment analysis, and protein-protein interaction (PPI) network analysis using the STRING database. This enabled the identification of candidate molecules potentially involved in SOX3 regulation, mitochondrial function maintenance, or epigenetic modification, including PRMT5, MTHFD2, and SIRT5.

### 2.37 Statistical analysis

Statistical analyses were conducted using GraphPad Prism 9 and R (v4.3). All tests were two-tailed with a significance threshold set at α = 0.05. Data normality and homogeneity of variance were assessed using the Shapiro-Wilk and Levene tests, respectively. For comparisons between two groups, an independent samples *t*-test was used (with Welch's correction if variance was unequal), or the Mann-Whitney *U* test if normality was not met. Comparisons among three or more groups were performed using one-way analysis of variance (ANOVA) (with Tukey or Šidák post hoc tests) or the Kruskal-Wallis test (with Dunn's post hoc). Two-way ANOVA or repeated-measures ANOVA was used for time-dependent or multifactorial data. When appropriate, linear mixed-effects models (lme4 package) were applied to account for missing data or unequal spacing, with individual subjects treated as random effects. Multiple testing corrections and multi-omics differential analysis were adjusted using the Benjamini-Hochberg FDR method. Outliers were identified and processed using ROUT or Grubbs' test at a predefined α level, with transparent reporting. Sample size estimation was performed using G*Power, indicating that n = 10 per group was sufficient to detect an effect size of f ≈ 0.4 with a power of 0.8 at α = 0.05 under a one-way ANOVA design. Statistical significance in graphs was denoted as ns, *, **, or *** for *p* ≥ 0.05, <0.05, <0.01, and <0.001, respectively. All analyses adhered to a pre-registered statistical analysis plan and were conducted under blinded conditions.

## 3 Results

### 3.1 TGF-β1 treatment significantly enhances TGF-βR density and functional modification of chondrocyte-derived exosomes

**Fig. 1:**
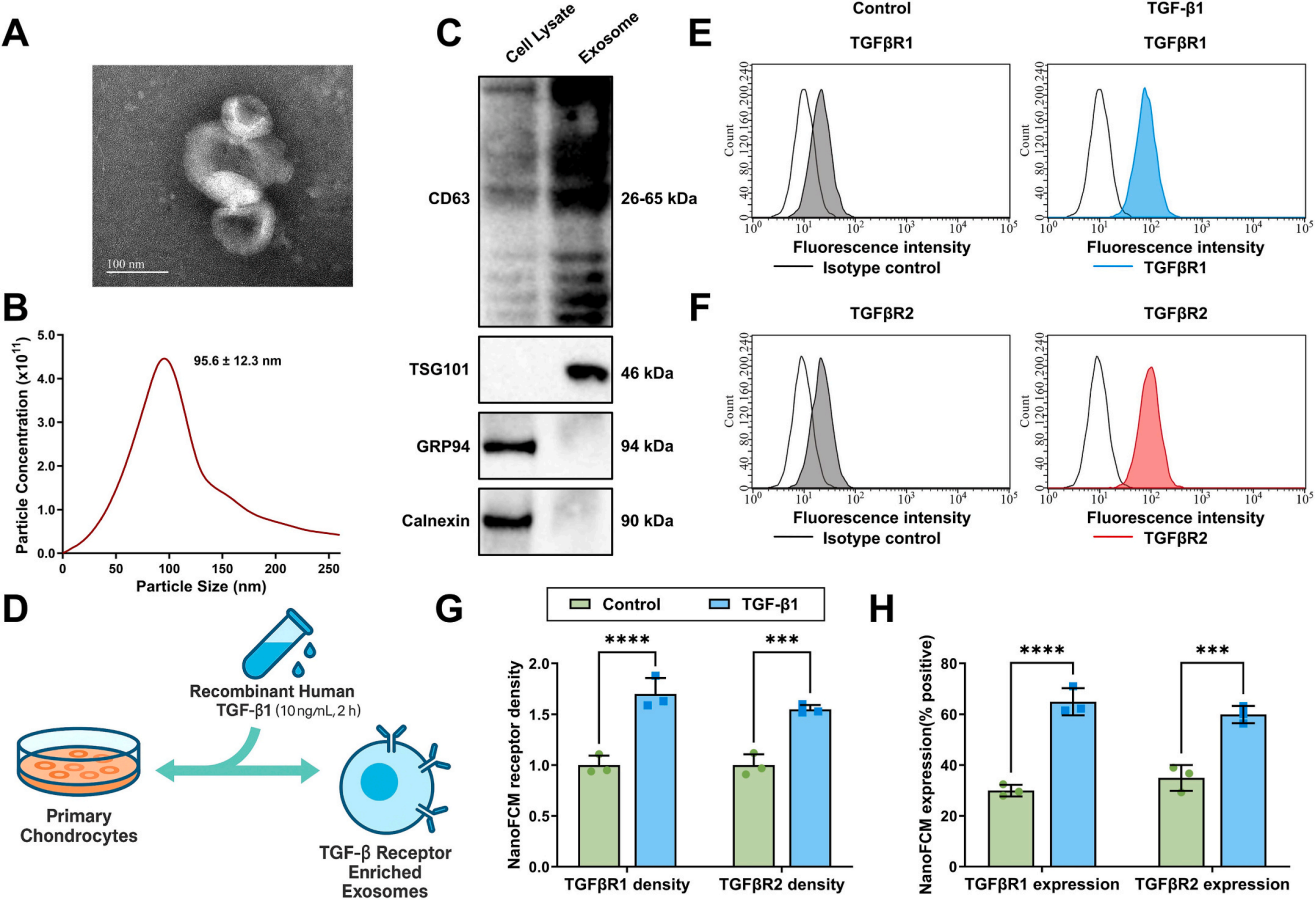
Validation of Exosome Purity, Characterization, and Targeting Modification Note: (A) TEM revealing the morphology and membrane structure of exosomes; scale bar = 100 nm. (B) NTA is assessing the size distribution and mean particle diameter of exosomes. (C) WB analysis of exosomal marker proteins CD63, TSG101, GRP94, and the negative control Calnexin. (D) Schematic representation of the experimental procedure for primary SD rat chondrocytes stimulated with TGF-β1 (10 ng/mL, 2 h). (E-F) Flow cytometry detection of TGF-βR1 and TGF-βR2 surface expression on exosomes. (G-H) NanoFCM-based single-particle analysis of expression density and enrichment levels of TGF-βR1 and TGF-βR2. All experiments were independently repeated at least three times. ***p < 0.001, ****p < 0.0001.

Chondrocyte-derived exosomes were first subjected to comprehensive characterization. Morphological assessment by TEM revealed a typical cup-shaped, bilayered vesicular structure with uniform size and minimal contaminants (Fig. 1A). NTA indicated that particle sizes predominantly ranged from 30 to 150 nm, consistent with classical exosomal profiles (Fig. 1B). WB analysis confirmed the presence of canonical exosomal markers CD63 and TSG101, while the absence of GRP94 and Calnexin indicated minimal contamination and high purity (Fig. 1C).

To generate functionally modified exosomes enriched with TGF-βRs, third-passage primary chondrocytes isolated from SD rats were cultured until reaching optimal adherence and then stimulated with recombinant human TGF-β1 (10 ng/mL) for 2 h to induce enrichment of TGF-βR1/R2 on the surface of the secreted exosomes (Fig. 1D). Flow cytometry analysis demonstrated a marked increase in TGF-βR1 and TGF-βR2 signals on the surface of exosomes derived from the TGF-β1-treated group compared to the untreated control (Fig. 1E–F). This receptor enrichment was further validated using NanoFCM single-particle analysis, which confirmed significantly higher receptor density and expression levels in the TGF-β1 group (Fig. 1G–H). Collectively, these findings verify that the exosomes exhibited high purity and well-defined characterization, and that functional surface modification targeting TGF-βRs was successfully achieved.

### 3.2 TGF-β1-stimulated exosomes form a stable and efficient composite system with PLGA nanoparticles

**Fig. 2:**
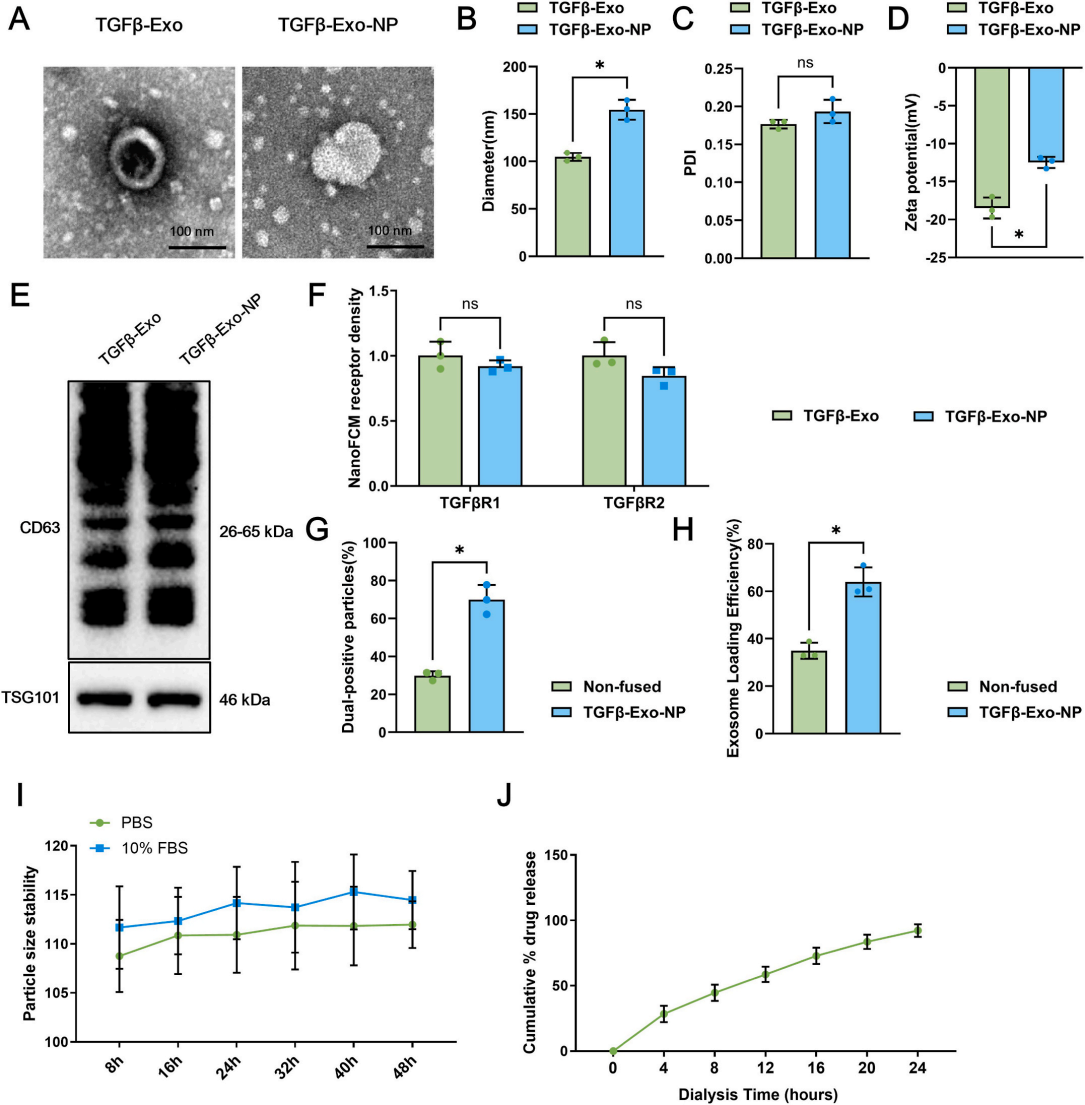
Characterization of the TGFβ-Exo-NP System Note: (A) TEM images of TGFβ-Exo and TGFβ-Exo-NP showing morphological structure; scale bar = 100 nm. (B) DLS measurement of particle diameter. (C) DLS assessment of PDI. (D) Zeta potential analysis. (E) WB detection of exosomal markers CD63 and TSG101. (F) NanoFCM single-particle analysis of TGFβR1 and TGFβR2 expression. (G) NanoFCM quantification of the proportion of particles double-positive for membrane markers and fluorescent nanoparticle labeling. (H) BCA protein quantification is used to calculate exosome loading efficiency. (I) Particle size stability of TGFβ-Exo-NP in PBS and 10% FBS over 48 h. (J) Cumulative release profile of TGFβ-Exo-NP over 24 h. All experiments were performed in triplicate or more. ns: not significant, *p < 0.05.

To evaluate the physicochemical properties and structural stability of receptor-enriched exosomes after complex formation with PLGA nanoparticles, the TGFβ-Exo-NP system was systematically characterized. TEM revealed that TGFβ-Exo exhibited a typical cup-shaped, bilayer membrane structure, whereas TGFβ-Exo-NP retained an overall vesicle-like morphology after complexation with PLGA nanoparticles. A continuous region of high electron density was observed along the particle periphery, indicating the formation of a tight and stable composite structure between exosomes and nanoparticles. The particles displayed uniform morphology, and no obvious aggregation was detected (Fig. 2A).

DLS analysis showed that the mean particle diameter increased from approximately 104.6 ± 7.9 nm for exosomes to 138.2 ± 12.1 nm after complex formation, while the polydispersity index (PDI) remained within the range of 0.18-0.22, indicating favorable dispersion and size uniformity of the composite system (Fig. 2B–C). The zeta potential shifted from −18.7 ± 1.4 mV for exosomes to −12.3 ± 1.7 mV following PLGA incorporation, suggesting a detectable alteration in surface charge after complexation (Fig. 2D).

The stability of exosomal membrane proteins during the complexation process was further examined. WB analysis demonstrated that the exosomal marker proteins CD63 and TSG101 were stably expressed in both the TGFβ-Exo and TGFβ-Exo-NP groups, with no apparent reduction in signal intensity (Fig. 2E). In parallel, TGF-βR1 and TGF-βR2 remained enriched after complex formation, indicating that the receptor-modification features induced by TGF-β1 stimulation were preserved during assembly of the composite system (Fig. 2F). NanoFCM single-particle analysis further revealed substantial overlap between the CD63 signal and the fluorescence signal of PLGA nanoparticles, with dual-positive particles accounting for 76.4% ± 4.9% of the total population, supporting the formation of a high proportion of stable exosome-nanoparticle complexes (Fig. 2G). In addition, exosome loading efficiency calculated based on BCA protein quantification showed that approximately 62.3% ± 5.1% of exosomal proteins were successfully associated with the PLGA nanoparticle system in TGFβ-Exo-NP (Fig. 2H).

Stability assessment further showed that TGFβ-Exo-NP maintained a stable particle size in both PBS and 10% FBS over 48 h (PBS: 108.77 ± 3.68 nm at 8 h and 111.97 ± 2.37 nm at 48 h; 10% FBS: 111.67 ± 4.20 nm at 8 h and 114.47 ± 2.97 nm at 48 h; Fig. 2I). Release profiling demonstrated a sustained release pattern, reaching 28.40 ± 6.30% at 4 h, 58.73 ± 5.86% at 12 h, and 92.23 ± 4.76% at 24 h (Fig. 2J).

Taken together, these results demonstrate that receptor-enriched exosomes derived from TGF-β1 stimulation form a stable composite system with PLGA nanoparticles at a high coupling efficiency.

### 3.3 TGFβ-Exo-NP achieved receptor-mediated efficient cellular uptake and nuclear-targeted delivery

**Fig. 3:**
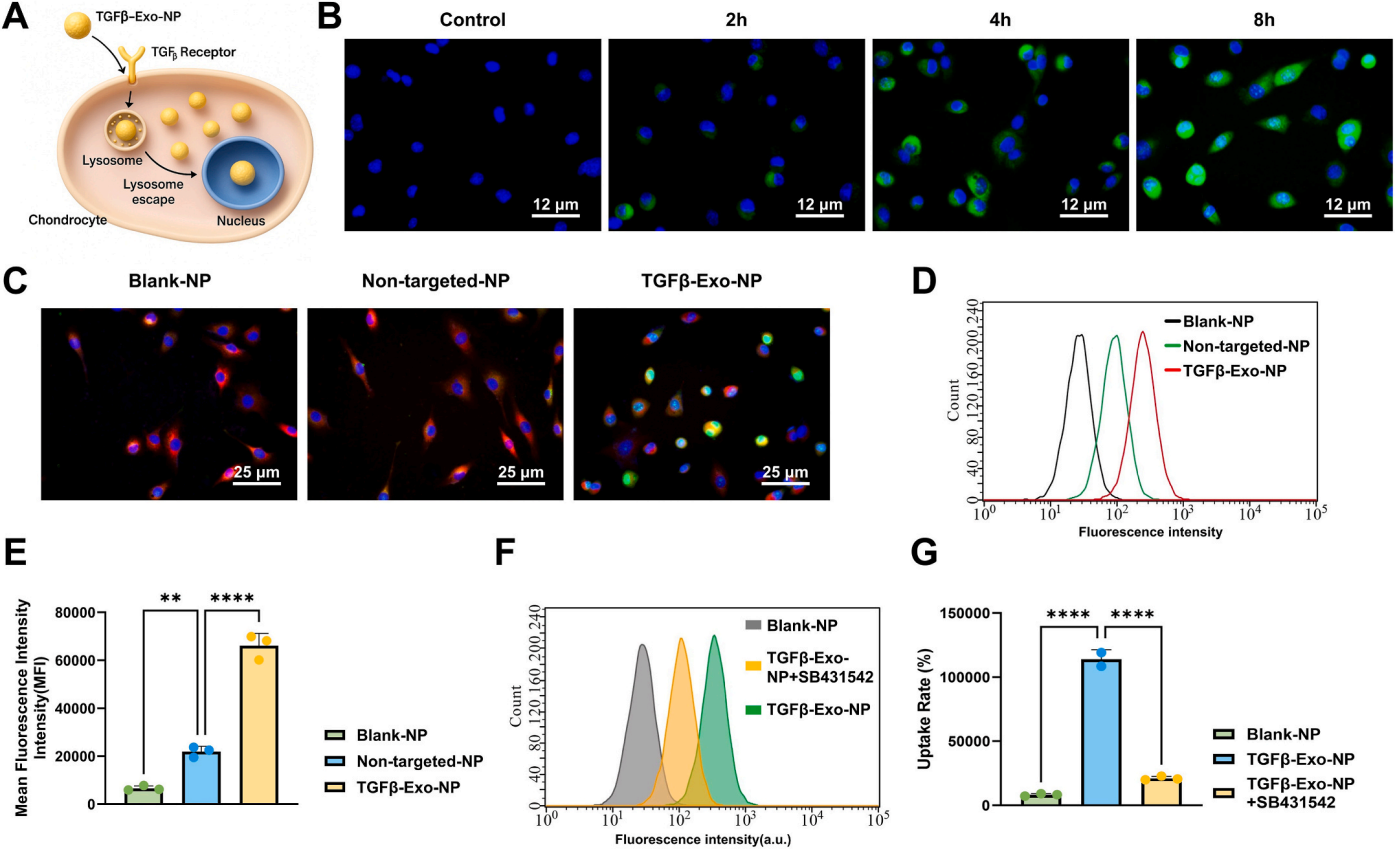
Analysis of TGFβ-Exo-NP-Mediated Cellular Uptake and Nuclear Localization Note: (A) Schematic illustration of the mechanism by which TGFβ-Exo-NP is specifically internalized by chondrocytes via TGF-βR-mediated binding and subsequently trafficked toward the nucleus. (B) Confocal microscopy images showing the intracellular localization of FITC-labeled TGFβ-Exo-NP at different incubation times (0, 2, 4, 8 h); green represents FITC signal, blue indicates nuclear DAPI staining; scale bar = 12 μm. (C) Confocal imaging of co-localization between FITC-TGFβ-Exo-NP (green) and LysoTracker-Red (red) to evaluate lysosomal escape; scale bar = 25 μm. (D-E) Flow cytometry analysis of MFI across different treatment groups. (F-G) Competitive inhibition assay using TGF-βR1-blocking antibody to assess changes in specific uptake efficiency. All experiments were independently repeated at least three times. Data are presented as mean ± SD. **p < 0.01, ****p < 0.0001.

To evaluate the delivery capability of TGFβ-Exo-NP, a FITC-labeled model was constructed and dynamically assessed using confocal microscopy and flow cytometry. We hypothesized that TGFβ-Exo-NP is efficiently internalized by chondrocytes through specific binding mediated by TGF-βRs, followed by lysosomal escape and entry into the cytoplasm and nucleus, thereby achieving effective nuclear delivery (Fig. 3A).

Time-course imaging revealed that intracellular green fluorescence became evident as early as 2 h post-incubation in the TGFβ-Exo-NP group, peaked at 8 h, and gradually expanded from the cytoplasmic region into the nucleus (Fig. 3B). Confocal imaging combined with LysoTracker staining further confirmed that the nanoparticles successfully escaped from lysosomes and partially localized to the nuclear compartment. In contrast, cells treated with Blank-NP (nanoparticles without exosome fusion) or Exo-NP (fused with unstimulated exosomes) exhibited weak fluorescence signals, predominantly restricted to the cytoplasm, with no evident nuclear localization (Fig. 3C).

Quantitative analysis by flow cytometry demonstrated that the MFI in the TGFβ-Exo-NP group was significantly higher than that of the Blank-NP and Exo-NP groups (Fig. 3D–E). To verify the receptor-dependent nature of TGFβ-Exo-NP cellular uptake, a competitive blockade experiment was performed using the TGF-βR kinase inhibitor SB431542. The results showed that, compared with the vehicle control group, pretreatment with SB431542 significantly reduced the uptake of TGFβ-Exo-NP by chondrocytes (Fig. 3F–G). These findings demonstrate that TGFβ-Exo-NP markedly enhances exosome delivery efficiency and enables effective nuclear-targeted localization.

### 3.4 TGFβ-Exo-NP significantly Suppressed Inflammatory cytokine expression and restored the chondrogenic phenotype

**Fig. 4:**
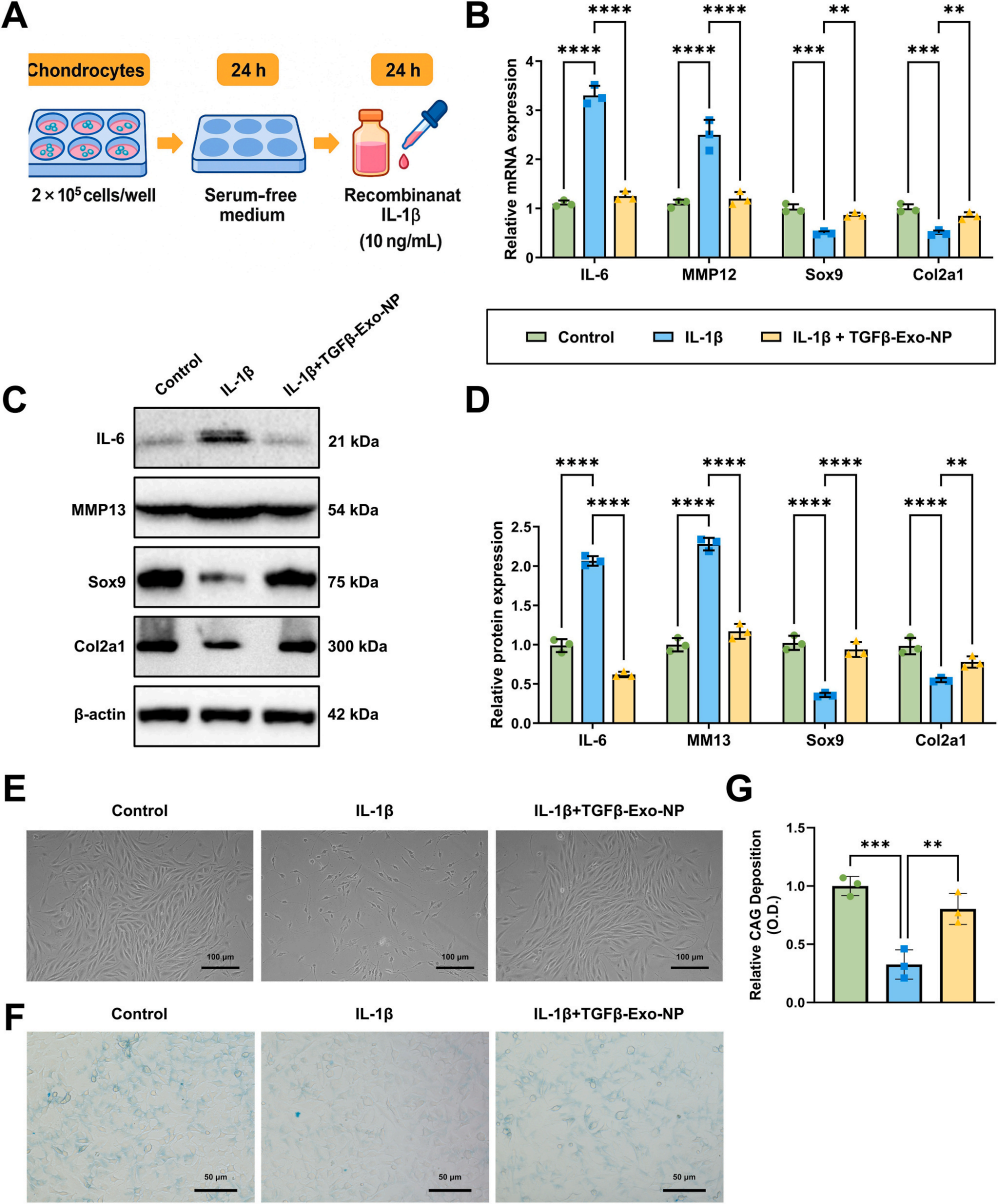
*In Vitro* Validation of TGFβ-Exo-NP in Suppressing Inflammation and Restoring Chondrocyte Phenotype Note: (A) Schematic diagram of the *in vitro* OA model: primary chondrocytes were seeded into 6-well plates, allowed to adhere for 24 h, then cultured in serum-free medium with rhIL-1β (10 ng/mL) stimulation for 24 h. (B) RT-qPCR analysis of mRNA expression levels of IL-6, MMP13, Sox9, and Col2a1. (C) WB detection of protein expression for IL-6, MMP13, Sox9, and Col2a1. (D) Densitometric quantification of WB bands normalized to β-actin. (E) Bright-field and phase-contrast microscopy images of chondrocyte morphology; scale bar = 100 μm. (F-G) Alcian Blue staining was performed to evaluate GAG deposition. All experiments were performed at least three times independently. Results are shown as mean ± SD. *p < 0.05, **p < 0.01, ****p < 0.0001.

To establish an *in vitro* inflammatory OA model, chondrocytes were seeded into 6-well plates at a density of 2 × 10^5^ cells/well. After 24 h of adherence, the medium was replaced with serum-free medium, followed by stimulation with recombinant human IL-1β (10 ng/mL) for 24 h (Fig. 4A). RT-qPCR (Fig. 4B) and WB analyses (Fig. 4C–D) revealed that IL-1β stimulation markedly induced inflammatory and degenerative responses, as evidenced by significant upregulation of IL-6 and MMP13 at both mRNA and protein levels, along with a notable decrease in the expression of chondrogenic markers Sox9 and Col2a1. Following treatment with TGFβ-Exo-NP, inflammatory activity and phenotypic deterioration in chondrocytes were substantially attenuated. Compared with the IL-1β group, cells treated with TGFβ-Exo-NP showed reduced IL-6 and MMP13 expression, while Sox9 and Col2a1 levels were significantly restored, approaching those of the unstimulated control group. Morphological evaluation further corroborated these findings. After IL-1β stimulation, chondrocytes exhibited characteristic OA-like changes, including cell shrinkage and reduced density. In contrast, TGFβ-Exo-NP treatment preserved cellular morphology, resulting in plumper cell bodies, firm adhesion, and enhanced intercellular connectivity. By comparison, cells in the IL-1β group appeared contracted, rounded, and sparsely distributed (Fig. 4E). To further functionally validate the protective effects of TGFβ-Exo-NP on the chondrocyte phenotype, Alcian Blue staining was performed on chondrocytes from different treatment groups to assess GAG deposition (Fig. 4F). The control group exhibited uniform and intense Alcian Blue-positive staining, indicating robust matrix synthetic capacity, whereas IL-1β stimulation markedly reduced staining intensity, suggesting that inflammatory stress impaired cartilage matrix synthesis. In contrast, treatment with TGFβ-Exo-NP significantly enhanced Alcian Blue staining in chondrocytes, with GAG deposition substantially restored to levels comparable to those of the unstimulated control group (Fig. 4F–G).

Taken together, these results demonstrated that TGFβ-Exo-NP effectively suppressed IL-1β-induced inflammation and restored the Sox9/Col2a1-mediated chondrogenic phenotype in an *in vitro* OA inflammation model.

### 3.5 Multi-omics integration reveals a SOX3-H3K4me3-linked cell death-related regulatory network

To comprehensively elucidate the role of TGFβ-Exo-NP in OA, multi-omics integration was performed using primary articular chondrocytes stimulated with IL-1β, comparing conditions with and without TGFβ-Exo-NP treatment. RNA-seq analysis identified 129 differentially expressed genes (DEGs), including 19 upregulated and 110 downregulated genes. The volcano plot clearly illustrated the distribution of significantly up- and downregulated genes (Fig. S1A). Subsequent GO enrichment analysis revealed that these DEGs were predominantly associated with functional modules such as cellular stress responses, peptidase complexes, caspase complexes, and chemokine receptor activity, indicating enhanced activation of inflammation- and cell death-related pathways (Fig. S1B). Notably, KEGG pathway network analysis revealed extensive interactions among multiple cell death-related pathways. These included apoptosis, necroptosis, TNF signaling, cytosolic DNA-sensing, IL-17 signaling, and chemokine signaling, which together formed a densely interconnected functional cluster (Fig. S1C). These findings suggest that under IL-1β stimulation, chondrocytes did not exclusively activate a single cell death pathway. Rather, they exhibited concurrent enrichment of several cell death-related pathways, including apoptosis, necroptosis, and pyroptosis-associated signaling.

TMT-based quantitative proteomics identified 64 DEPs (Fig. S1D). Among the upregulated proteins, epigenetic modifiers such as PRMT5, SMYD3, and WDR5 were significantly elevated, indicating an enhancement of chromatin activation marks. Concurrently, metabolic regulators, including ACAT2, were also upregulated, suggesting a reprogramming of cellular metabolic states. Notably, cell death-associated mediators, including MLKL, Caspase-8, and ZBP1, were also upregulated, suggesting activation of necroptotic and inflammatory cell death pathways. Importantly, TGFβ-Exo-NP treatment markedly suppressed several inflammation-amplifying immune mediators, including CCL20, NFKB1, and STAT1, indicating that this nanosystem exerted an anti-inflammatory effect on chondrocytes. Among the downregulated proteins, matrix-degrading enzymes associated with cartilage degeneration, such as MMP3 and MMP13, were significantly reduced, while autophagy-related protein LC3B showed a partial recovery trend (Fig. S1E). Functional enrichment analysis further revealed that these DEPs were significantly clustered in pathways related to FoxO signaling, apoptosis, necroptosis, TNF signaling, IL-17 signaling, and chemokine signaling—collectively forming a tightly connected cell death-related pathway network that mirrored the RNA-seq enrichment profile (Fig. S1F–G). These findings suggest that the epigenetic modification-energy metabolism-cell death-related axis constitutes the core regulatory mechanism through which TGFβ-Exo-NP exerts its effects.

Based on integrated analyses of RNA-seq and TMT-based quantitative proteomics, we first consistently observed significant upregulation of Sox family members at both the transcriptional and protein expression levels (Fig. S1A and D), suggesting that they may serve as key downstream effectors in TGFβ-Exo-NP-mediated regulation of the chondrocyte phenotype. Given that Sox genes involved in chondrogenesis and phenotype maintenance are highly dependent on promoter-level transcriptional regulation, we further employed ChIP-seq to systematically assess changes in their epigenetic status. The results showed that, compared with the control group, TGFβ-Exo-NP treatment markedly increased H3K4me3 occupancy at the Sox3 promoter region, accompanied by broader and more continuous enrichment peaks; in parallel, a coordinated global elevation of H3K4me3 signals was also observed across the Sox3 gene body (Fig. S1H).

By integrating RNA-seq, ChIP-seq, and proteomics data, we established an H3K4me3-dependent SOX3 regulatory network. The results indicate that SOX3 functions as a central node linking epigenetic modification with metabolic remodeling and cell fate determination, playing a pivotal role in the regulation of inflammation- and cell death-related pathways.

### 3.6 TGFβ-Exo-NP significantly upregulates SOX3 expression and activates H3K4me3 signaling

**Fig. 5:**
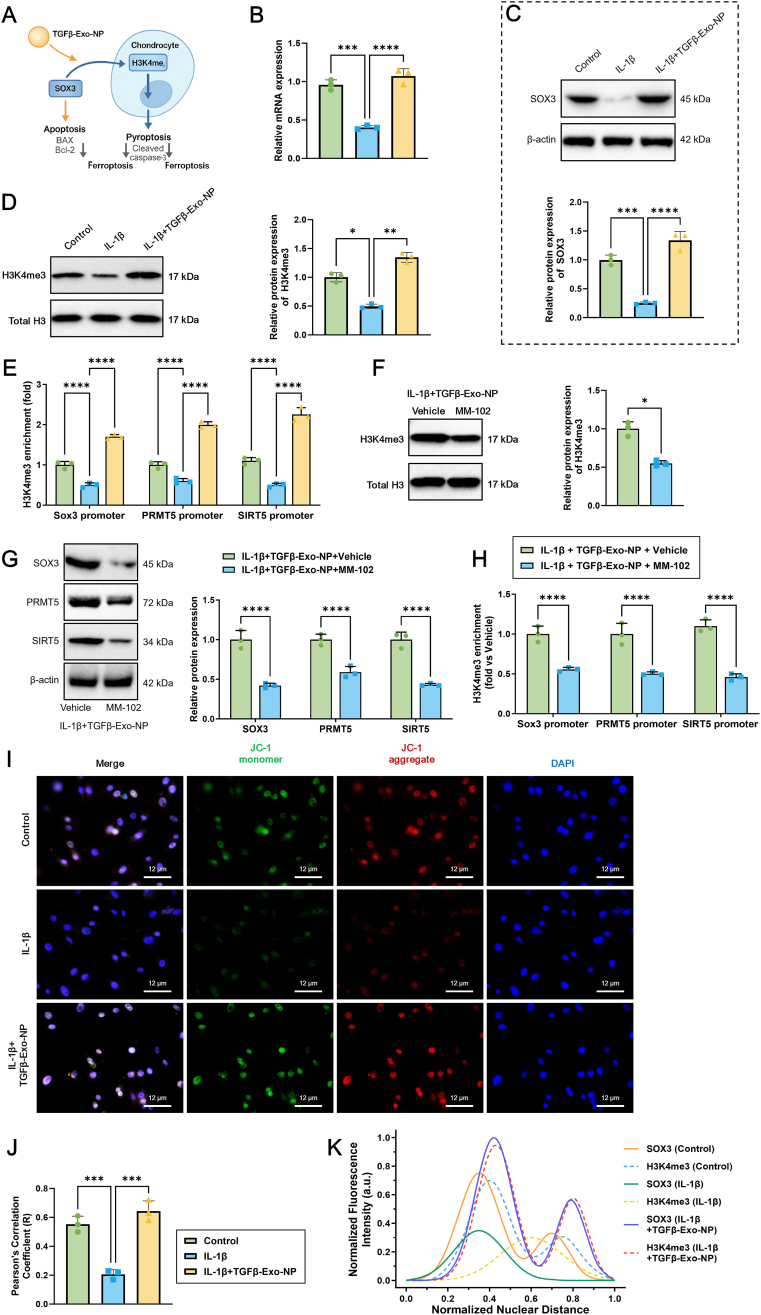
TGFβ-Exo-NP Upregulates SOX3 and Activates the H3K4me3 Epigenetic Pathway Note: (A) Schematic illustration of the proposed mechanism: following cellular uptake, TGFβ-Exo-NP promotes SOX3 upregulation and activates H3K4me3 modification. (B) RT-qPCR analysis of SOX3 mRNA expression. (C) WB analysis of SOX3 protein levels. (D) Acid-extracted total histone WB analysis of H3K4me3 and total H3. (E) ChIP-qPCR detection of H3K4me3 enrichment at the promoter regions of SOX3, PRMT5, and SIRT5. (F) WB detection of H3K4me3 and total H3 following MM-102 treatment. (G) WB analysis of PRMT5 and SIRT5 protein expression. (H) ChIP-qPCR analysis of H3K4me3 enrichment at the promoter regions of SOX3, PRMT5, and SIRT5. (I) Dual immunofluorescence staining combined with confocal microscopy was used to examine the intranuclear distribution and overlapping signals of SOX3 (green) and H3K4me3 (red); nuclei were counterstained with DAPI. Scale bar = 12 μm. (J) Quantitative analysis of SOX3 and H3K4me3 colocalization based on Pearson's correlation coefficient (*r* value). (K) Fluorescence intensity line-scan analysis (nuclear profile) illustrating the signal intensity distribution of SOX3 and H3K4me3. All experiments were independently repeated at least three times. *p < 0.05, **p < 0.01, ***p < 0.001, ****p < 0.0001.

To verify the regulatory effect of TGFβ-Exo-NP on downstream epigenetic modification signals, we performed analyses of mRNA and protein expression as well as signal correlation to determine whether TGFβ-Exo-NP upregulates SOX3 expression and activates H3K4me3 modification (Fig. 5A).

RT-qPCR and WB results confirmed that IL-1β stimulation markedly reduced both SOX3 mRNA and protein levels, whereas TGFβ-Exo-NP intervention significantly upregulated SOX3 expression (Fig. 5B–C). Concurrently, IL-1β stimulation led to a global reduction in H3K4me3 levels, while TGFβ-Exo-NP treatment substantially restored this modification (Fig. 5D), suggesting a functional coupling between SOX3 and epigenetic signaling. ChIP-qPCR revealed that H3K4me3 enrichment at the promoters of SOX3 target genes (PRMT5 and SIRT5) was significantly diminished in the IL-1β group, whereas TGFβ-Exo-NP markedly increased site-specific H3K4me3 enrichment at these loci compared to IL-1β alone (Fig. 5E).

Following treatment with the MLL1-specific inhibitor MM-102 to block H3K4me3 modification, WB analysis showed a global decline in H3K4me3 levels (Fig. 5F). Notably, inhibition with MM-102 also led to a concurrent reduction in the protein expression of the downstream metabolic regulators SOX3, PRMT5, and SIRT5 (Fig. 5G). ChIP-qPCR further confirmed that MM-102 treatment significantly attenuated site-specific H3K4me3 enrichment at the promoters of SOX3, PRMT5, and SIRT5 (Fig. 5H). These findings collectively indicate that TGFβ-Exo-NP exerts its regulatory effects by upregulating SOX3 and activating H3K4me3 modification.

To verify the spatial interaction between SOX3 and H3K4me3 within the nucleus, quantitative analysis was performed using dual immunofluorescence staining combined with confocal microscopy. The results showed that in the control group, SOX3 and H3K4me3 were primarily distributed along the nuclear periphery, with relatively weak signals and limited overlap. In the IL-1β group, the overall expression levels of both markers were markedly reduced and displayed a diffuse distribution pattern, indicating suppression of transcriptional activation under inflammatory conditions. In contrast, in the IL-1β + TGFβ-Exo-NP group, SOX3 and H3K4me3 signals were significantly enhanced and exhibited a high degree of nuclear colocalization (Fig. 5I–J), suggesting pronounced spatial coupling between the two following TGFβ-Exo-NP intervention.

Fluorescence intensity line-scan analysis further demonstrated overlapping peak signals of SOX3 and H3K4me3 in both the perinuclear and central nuclear regions in the TGFβ-Exo-NP group, with signal intensities significantly higher than those observed in the other two groups (Fig. 5K), indicating that epigenetic regulation is closely associated with intranuclear spatial colocalization.

### 3.7 Untargeted omics reveal significant remodeling of metabolic pathways

To systematically evaluate the regulatory effects of TGFβ-Exo-NP on IL-1β-induced energy metabolism dysregulation, we conducted comprehensive untargeted metabolomic profiling using LC-MS. The volcano plot revealed substantial differences in metabolite levels between the two groups, including marked alterations in key metabolites involved in purine metabolism and the tricarboxylic acid (TCA) cycle, such as adenine, succinate, and fumarate (Fig. S2A). PLS-DA further demonstrated a clear separation along the first principal component, indicating that exosome treatment induced global remodeling of the metabolic network (Fig. S2B). VIP analysis identified adenine, succinate, fumarate, succinyl-CoA, GSSG, cis-aconitate, GSH, malate, and AMP as the primary contributors to intergroup differentiation (Fig. S2C). The heatmap analysis further showed that TGFβ-Exo-NP significantly suppressed IL-1β-induced accumulation of purine degradation products (adenine, adenosine, AMP, hypoxanthine) and inflammatory intermediates such as succinate and fumarate, while concurrently upregulating mitochondrial and antioxidative metabolites, including succinyl-CoA, malate, and GSH (Fig. S2D).

Metabolic pathway network analysis revealed that the differential metabolites were primarily clustered around the TCA cycle as a central hub, exhibiting close interconnections with glyoxylate metabolism, pyruvate metabolism, amino acid metabolism, purine metabolism, and glutathione metabolism (Fig. S2E). These findings suggest that exosome treatment reshaped a multi-pathway energy network centered on the TCA cycle. Functional assays corroborated the omics data: TGFβ-Exo-NP significantly reduced lactate accumulation (Fig. S2F) and elevated the NAD^+^/NADH ratio (Fig. S2G), further confirming that exosome intervention restored mitochondrial oxidative capacity and improved cellular redox homeostasis.

### 3.8 TGFβ-Exo-NP enhances mitochondrial membrane potential and activity to restore energy metabolism

**Fig. 6:**
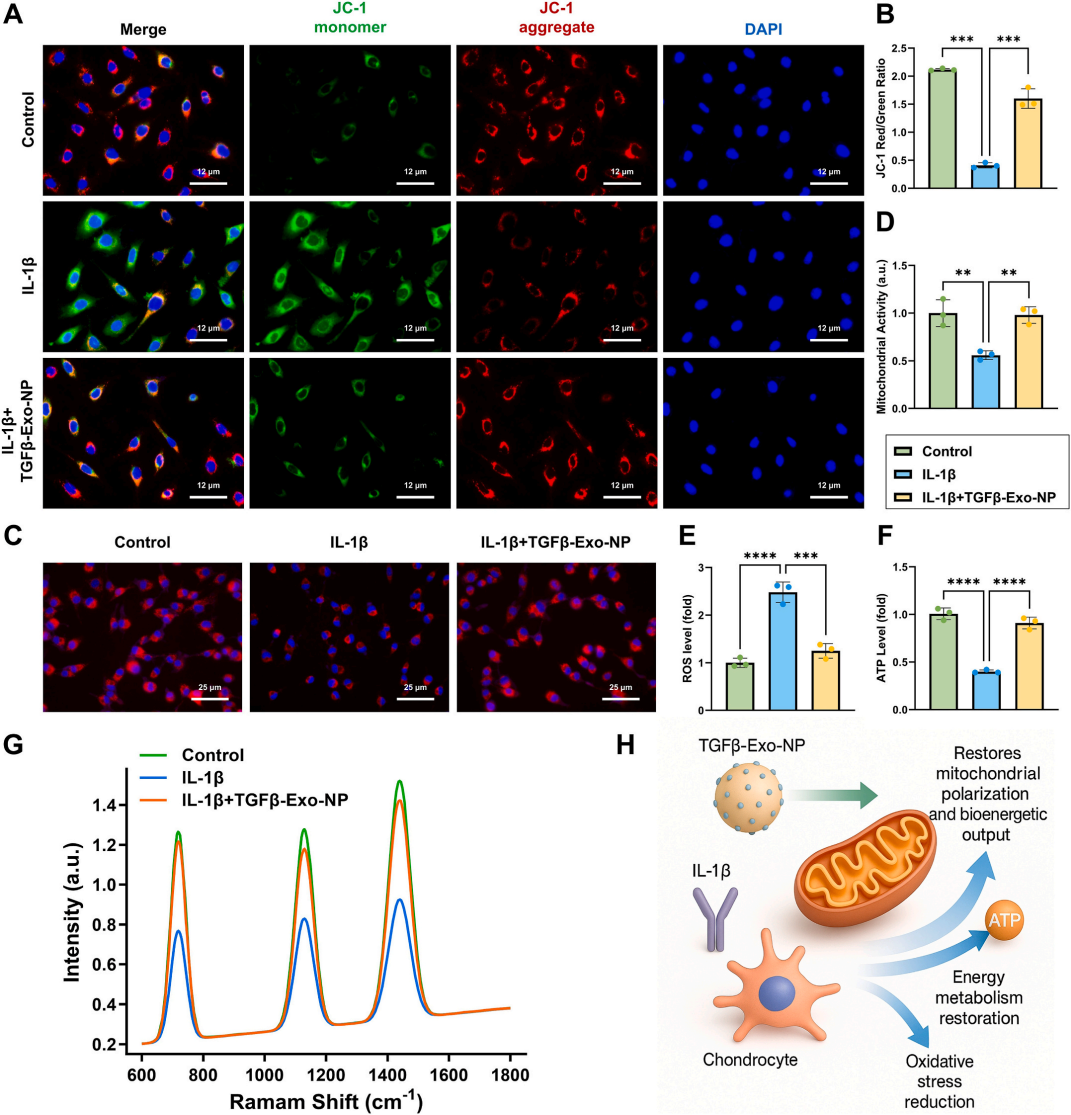
Multilevel Analysis of TGFβ-Exo-NP in Improving Mitochondrial Membrane Potential, Structure, and Energy Metabolism Note: (A) JC-1 staining to assess changes in mitochondrial membrane potential; the red/green fluorescence ratio reflects the degree of polarization. Scale bar = 12 μm. (B) Quantitative analysis of the red/green JC-1 fluorescence ratio. (C) MitoTracker Red staining to visualize mitochondrial morphology. Scale bar = 25 μm. (D) Quantitative analysis of mitochondrial morphological parameters. (E) ROS levels were assessed using a ROS-sensitive probe. (F) Cellular ATP levels were quantified using an ATP colorimetric assay kit. (G) Raman spectroscopy to detect changes in the intensity of energy metabolism-specific peaks (720, 1130, 1440 cm^−1^). (H) Schematic illustration summarizing the mechanism by which TGFβ-Exo-NP regulates mitochondrial function and energy metabolism. All experiments were repeated at least three times. **p < 0.01, ***p < 0.001, ****p < 0.0001.

To evaluate the regulatory effects of TGFβ-Exo-NP on mitochondrial function and energy metabolism, we employed a combination of multi-level imaging and spectroscopic analyses. JC-1 staining revealed that, compared to the Control group, IL-1β treatment significantly reduced the red-to-green fluorescence ratio, indicating impaired mitochondrial membrane potential. In contrast, TGFβ-Exo-NP intervention markedly increased this ratio, suggesting a restoration of mitochondrial polarization capacity (Fig. 6A–B). MitoTracker staining further demonstrated that mitochondria in the IL-1β group exhibited fragmented and disrupted morphology, whereas those in the IL-1β + TGFβ-Exo-NP group displayed elongated and continuous filamentous structures, with markedly improved structural integrity (Fig. 6C–D).

ROS probe assays showed a substantial increase in intracellular ROS levels following IL-1β stimulation, which was significantly attenuated by TGFβ-Exo-NP treatment (Fig. 6E), indicating effective alleviation of mitochondrial oxidative stress. Quantitative ATP measurements revealed a pronounced reduction in intracellular ATP content in the IL-1β group, whereas TGFβ-Exo-NP intervention significantly restored ATP levels, approaching those of the control (Fig. 6F), further confirming the recovery of energy production capacity. Raman spectroscopy detected characteristic energy metabolism peaks (720, 1130, 1440 cm^−1^), which were significantly enhanced after TGFβ-Exo-NP treatment, indicating improved oxidative phosphorylation and energy output (Fig. 6G). Collectively, these results demonstrate that TGFβ-Exo-NP effectively reverses IL-1β-induced energy metabolism impairment in chondrocytes by restoring mitochondrial membrane potential and functional activity (Fig. 6H).

### 3.9 TGFβ-Exo-NP intervention attenuates apoptosis-, pyroptosis-, and ferroptosis-related pathways

**Fig. 7:**
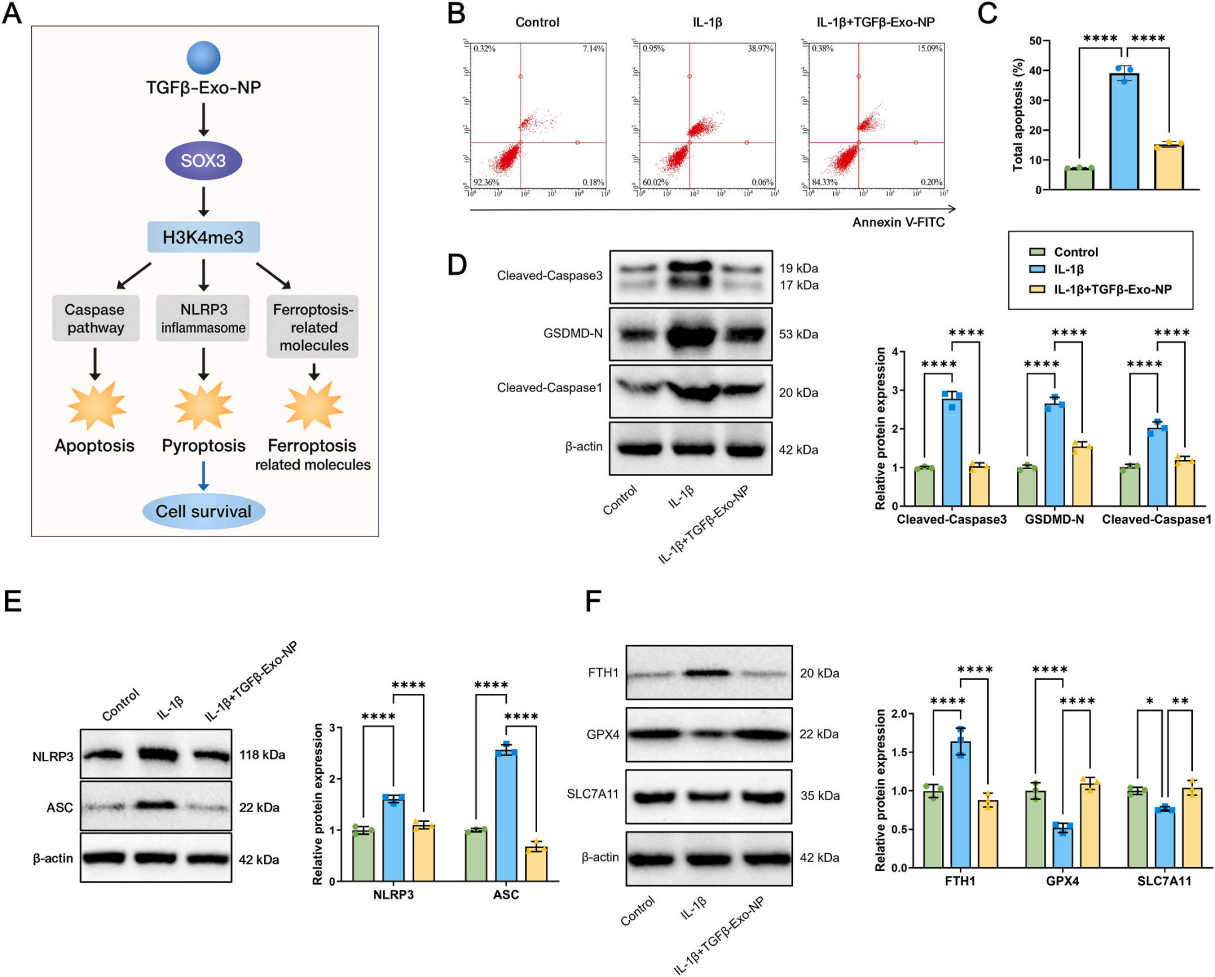
TGFβ-Exo-NP intervention attenuates apoptosis-, pyroptosis-, and ferroptosis-related changes through the SOX3/H3K4me3 axis. Note: (A) Schematic illustration showing that TGFβ-Exo-NP activates the SOX3/H3K4me3 signaling axis to coordinately inhibit apoptosis, pyroptosis, and ferroptosis pathways. (B-C) Flow cytometry using Annexin V/PI double staining to quantify chondrocyte apoptosis. (D) WB analysis of apoptosis-related protein cleaved caspase-3 and pyroptosis-related proteins GSDMD-N and cleaved caspase-1. (E) WB analysis of inflammasome-related proteins NLRP3 and ASC. (F) WB analysis of ferroptosis-associated molecules GPX4, SLC7A11, and FTH1. All experiments were independently repeated at least three times. ****p < 0.0001.

To comprehensively evaluate the regulatory effects of TGFβ-Exo-NP on IL-1β-induced apoptosis-, pyroptosis-, and ferroptosis-related changes in chondrocytes, we assessed apoptosis-, pyroptosis-, and ferroptosis-related markers (Fig. 7A).

Flow cytometry analysis revealed that IL-1β stimulation markedly increased the total apoptosis rate compared with the Control group. In contrast, TGFβ-Exo-NP treatment significantly reduced the apoptotic cell population, indicating potent suppression of apoptosis signaling (Fig. 7B–C). WB analysis further confirmed that IL-1β exposure led to pronounced upregulation of the apoptotic execution protein cleaved caspase-3, as well as pyroptosis-related proteins GSDMD-N and cleaved caspase-1. These increases were significantly attenuated following TGFβ-Exo-NP treatment (Fig. 7D). Additionally, inflammasome pathway analysis showed that NLRP3 and ASC were strongly activated in the IL-1β group, whereas their upregulation was markedly suppressed by TGFβ-Exo-NP (Fig. 7E), suggesting effective inhibition of both apoptotic and pyroptotic pathways. Ferroptosis analysis demonstrated that IL-1β stimulation led to significant downregulation of anti-ferroptotic proteins GPX4 and SLC7A11, alongside upregulation of the ferroptosis marker FTH1. Notably, TGFβ-Exo-NP treatment reversed these trends, restoring GPX4 and SLC7A11 expression and reducing FTH1 levels (Fig. 7F), indicating a recovery of redox balance and mitigation of lipid peroxidation and iron overload.

Collectively, these findings demonstrate that TGFβ-Exo-NP attenuates apoptosis-, pyroptosis-, and ferroptosis-related molecular changes through the SOX3/H3K4me3 signaling axis, thereby protecting chondrocytes from inflammation-induced damage.

### 3.10 SOX3 serves as a critical regulatory node in TGFβ-Exo-NP-mediated chondrocyte homeostasis and cytoprotection

**Fig. 8:**
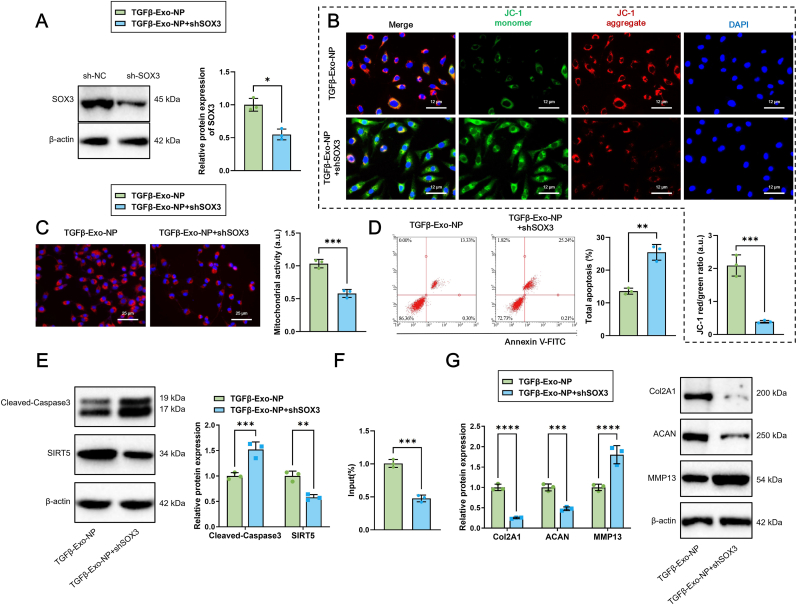
Mechanism-Dependent Role of the Key Target SOX3 Validated by Functional Interventions Note: (A) WB analysis verifying the knockdown efficiency of SOX3. (B) JC-1 fluorescence staining to evaluate changes in mitochondrial membrane potential. Scale bar = 12 μm. (C) MitoTracker Red fluorescence staining to assess mitochondrial morphology and distribution. Scale bar = 25 μm. (D) Flow cytometry using Annexin V/PI double staining to quantify apoptotic cell populations. (E) WB analysis of cleaved caspase-3 and SIRT5 protein expression. (F) ChIP-qPCR analysis detecting SOX3 enrichment at the SIRT5 promoter. (G) WB analysis of cartilage matrix-related proteins, including Col2a1, Acan, and MMP13. All experiments were independently repeated at least three times. **p < 0.01, ***p < 0.001, ****p < 0.0001.

To determine the pivotal role of SOX3 in TGFβ-Exo-NP-mediated regulation of mitochondrial homeostasis and apoptosis suppression, a shSOX3 model was established (Fig. 8A) and subjected to TGFβ-Exo-NP treatment (TGFβ-Exo-NP vs. TGFβ-Exo-NP + shSOX3). JC-1 staining revealed that cells in the TGFβ-Exo-NP group exhibited a pronounced increase in red J-aggregate fluorescence and a corresponding decrease in green monomer signal, resulting in a significantly elevated red/green fluorescence ratio, indicative of restored and highly polarized mitochondrial membrane potential. However, upon shSOX3 treatment (TGFβ-Exo-NP + shSOX3), the red fluorescence was substantially reduced while the green signal intensified, leading to a decreased red/green ratio, thereby suggesting that shSOX3 impaired the mitochondrial polarization capacity restored by TGFβ-Exo-NP (Fig. 8B). Further MitoTracker Red staining demonstrated that TGFβ-Exo-NP promoted elongated, filamentous, and interconnected mitochondrial networks with preserved structural integrity. In contrast, the shSOX3 group exhibited severe mitochondrial fragmentation, punctate aggregation, disrupted networks, and reduced density (Fig. 8C).

Flow cytometric Annexin V/PI staining confirmed apoptotic changes: the TGFβ-Exo-NP group displayed low proportions of both early and late apoptotic cells, with a total apoptosis rate of approximately 13%. This rate significantly increased in the TGFβ-Exo-NP + shSOX3 group, indicating that shSOX3 markedly diminished the anti-apoptotic effects of TGFβ-Exo-NP (Fig. 8D). WB analysis further supported these findings: cleaved caspase-3 expression was downregulated, and SIRT5 was upregulated in the TGFβ-Exo-NP group, suggesting suppressed apoptosis and enhanced metabolic function. However, shSOX3 reversed these changes, with increased cleaved caspase-3 and reduced SIRT5 levels, implying a loss of the protective function of the SOX3 signaling axis (Fig. 8E). Moreover, ChIP-qPCR confirmed that SOX3 was directly enriched at the SIRT5 promoter, thereby regulating its expression (Fig. 8F). At the matrix metabolism level, the TGFβ-Exo-NP group showed elevated expression of matrix synthesis markers Col2a1 and Acan, and decreased expression of the catabolic marker MMP13. These effects were reversed upon shSOX3, with reduced Col2a1 and Acan and increased MMP13 expression, indicating that SOX3 is also involved in matrix homeostasis regulated by TGFβ-Exo-NP (Fig. 8G).

Taken together, TGFβ-Exo-NP maintained mitochondrial membrane potential and structural integrity by activating the SOX3 signaling axis, enhanced SIRT5-mediated metabolic function, and promoted matrix synthesis while suppressing chondrocyte apoptosis.

### 3.11 TGFβ-Exo-NP significantly alleviates OA symptoms via SOX3

First, to validate the structural and functional effectiveness of the knee OA animal model established by DMM surgery, preliminary pathological evaluation was performed using small-animal computed tomography on postoperative day 7. The results showed that rats in the DMM model group exhibited pronounced medial meniscus displacement accompanied by increased intra-articular fluid accumulation, indicating postoperative joint instability and activation of inflammatory responses (Fig. S3A). In contrast, no such abnormalities were observed in the Sham group, in which joint architecture remained intact, confirming that the surgical intervention was the primary causative factor. Subsequently, gait analysis was conducted on postoperative days 14 and 28 to assess changes in locomotor behavior. Animals in the model group displayed marked gait disturbances, including reductions in mean stride width, stride length, and toe spread, indicating joint dysfunction resulting from cartilage degeneration (Fig. S3B–D). In addition, knee joint circumference in the model group remained persistently increased after surgery and was significantly greater than that in the Sham group, reflecting local swelling and joint effusion (Fig. S3E). Forelimb grip strength testing further demonstrated a decline in muscle strength in the model group on postoperative day 28, suggesting compromised joint load-bearing capacity (Fig. S3F).

To further validate the therapeutic potential of TGFβ-Exo-NP in an OA animal model, rats received intra-articular injections for 4 consecutive weeks starting on postoperative day 7 following DMM surgery (DMM, DMM + TGFβ-Exo-NP, and TGFβ-Exo-NP + shSOX3 groups).

At the tissue level, H&E staining showed intact histological architecture in the heart, liver, spleen, lung, and kidney, with no obvious cellular necrosis, edema, or inflammatory cell infiltration (Fig. S4A). To further assess the systemic toxicity of TGFβ-Exo-NP, serum samples were collected from mice in each group for biochemical analysis. The results showed that alanine aminotransferase (ALT), aspartate aminotransferase (AST), blood urea nitrogen (BUN), and creatinine (Cr) levels remained within the normal physiological range and did not differ significantly from those in the Sham group (p > 0.05) (Fig. S4B). Together, these findings demonstrate that TGFβ-Exo-NP did not cause evident organ toxicity and exhibited favorable biosafety and translational potential.

Gait behavioral assessments revealed that the TGFβ-Exo-NP group exhibited significant functional improvement on postoperative day 28, whereas this effect was partially attenuated when SOX3 was simultaneously knocked down. Specifically, gait analysis showed that mean stride width, stride length, and toe spread in the TGFβ-Exo-NP group were markedly increased compared with those in the DMM model group and approached normal gait levels. In contrast, these gait parameters were significantly reduced in the TGFβ-Exo-NP + shSOX3 group relative to the TGFβ-Exo-NP group, indicating that the therapeutic effect depended on SOX3 activation (Fig. S3G–J). Forelimb grip strength testing further confirmed that TGFβ-Exo-NP treatment significantly enhanced joint-associated muscle strength and load-bearing capacity, whereas shSOX3 weakened this improvement (Fig. S3K). Collectively, these findings demonstrate that TGFβ-Exo-NP exhibits efficient *in vivo* delivery and robust cartilage reparative effects, which are functionally dependent on SOX3-mediated activation (Fig. S3L).

**Fig. 9:**
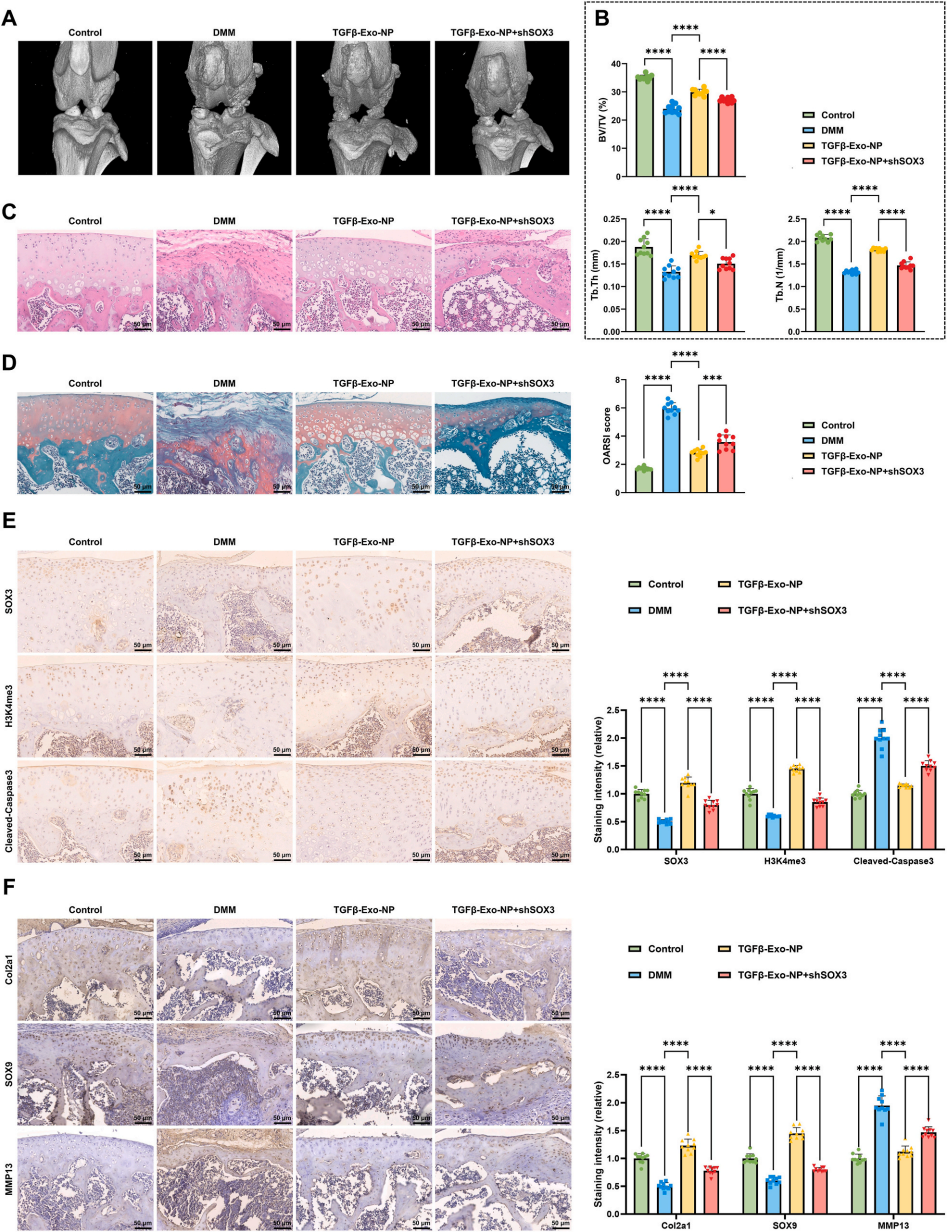
TGFβ-Exo-NP Attenuates Cartilage Degradation and Subchondral Bone Structural Damage Note: (A) Micro-CT 3D reconstruction of the knee joint to visualize subchondral bone architecture. Scale bar = 1 mm. (B) Quantitative analysis of BV/TV, Tb.Th, and Tb.N. (C) H&E staining to assess cartilage structural integrity and chondrocyte arrangement. Scale bar = 50 μm. (D) Safranin O/Fast Green staining to evaluate cartilage matrix composition and OARSI scoring. Scale bar = 50 μm. (E) IHC to examine the expression and distribution of SOX3, H3K4me3, and cleaved caspase-3 in cartilage tissue. Scale bar = 50 μm. (F) IHC analysis of Col2a1, SOX9, and MMP13 expression and distribution in cartilage tissue; scale bar = 50 μm. N = 10, *p < 0.05, ***p < 0.001, ****p < 0.0001.

To further evaluate the protective effects of TGFβ-Exo-NP at the tissue level, multimodal histological and morphological analyses were performed on knee joints from each experimental group. Three-dimensional micro-CT reconstruction revealed that, compared with the Sham group, the DMM group exhibited pronounced subchondral trabecular sparsity and disruption, accompanied by significant reductions in BV/TV, Tb.Th, and Tb.N. Following TGFβ-Exo-NP treatment, trabecular continuity and density were markedly improved, with significant increases in BV/TV, Tb.Th, and Tb.N. In contrast, these improvements were attenuated in the TGFβ-Exo-NP + shSOX3 group, indicating that SOX3 downregulation weakened the protective effects on bone microarchitecture (Fig. 9A–B).

H&E and Safranin O/Fast Green staining showed that the DMM group displayed a rough cartilage surface, disorganized chondrocyte arrangement, reduced matrix staining, and elevated OARSI scores. In contrast, TGFβ-Exo-NP treatment resulted in a smoother cartilage surface, restoration of cellular organization, enhanced matrix staining, and a significant reduction in OARSI scores. However, only partial recovery was observed in the TGFβ-Exo-NP + shSOX3 group, accompanied by compromised cartilage integrity (Fig. 9C–D). IHC analysis further demonstrated that TGFβ-Exo-NP markedly upregulated SOX3 expression in cartilage tissue, along with enhanced H3K4me3 labeling. In parallel, the DMM group exhibited a pronounced increase in cleaved caspase-3-positive cells, whereas this apoptotic signal was substantially reduced following TGFβ-Exo-NP treatment; notably, a rebound increase was observed upon shSOX3, suggesting that SOX3 downregulation partially reversed the anti-apoptotic effects of TGFβ-Exo-NP (Fig. 9E). Meanwhile, the positive signals of Col2a1 and SOX9 in the cartilage matrix and nuclei were significantly elevated, whereas expression of the matrix degradation-associated protein MMP13 was markedly reduced. Conversely, in the shSOX3 co-treatment group, recovery of Col2a1 and SOX9 expression was notably constrained, and MMP13 expression was upregulated again, indicating that SOX3 downregulation disrupted the TGFβ-Exo-NP-mediated balance between cartilage synthesis and degradation (Fig. 9F).

Taken together, these findings demonstrate that TGFβ-Exo-NP confers multidimensional protection against DMM-induced OA at both the tissue and bone microstructural levels by activating the SOX3-H3K4me3 epigenetic regulatory axis, whereas SOX3 downregulation compromises its integrated effects on bone microarchitecture repair, cartilage integrity maintenance, and apoptosis suppression.

### 3.12 Exosomal proteomics reveals that TGFβ-exo-NP is enriched in WDR5 and other epigenetic and metabolic regulators

To characterize the functional protein components of TGFβ-Exo-NP, a systematic LC-MS/MS proteomic profiling was conducted. A total of 92 exosomal proteins were identified, among which epigenetic and chromatin regulatory molecules such as WDR5, SMYD3, and PRMT5 were markedly upregulated. In addition, significant alterations were observed in energy metabolism-related proteins, including MTHFD2 and PDK1 (Fig. S5A–B). These molecules are closely associated with epigenetic regulation, mitochondrial metabolism, and cell death control. GO enrichment analysis revealed that the DEPs were significantly enriched in biological processes such as “protein methyltransferase complex,” “chromatin regulation,” and “metabolic stress response,” underscoring the central role of chromatin reprogramming in the functional effects of TGFβ-Exo-NP. KEGG pathway analysis further indicated that these proteins were concentrated in key metabolic and cell fate pathways, including “lysine degradation,” “tryptophan metabolism,” “cell cycle,” and the “FoxO signaling pathway,” suggesting that mitochondrial metabolic remodeling and cell cycle regulation may occur in a coordinated manner (Fig. S5C–D), and that exosomes may act through a metabolism-cell death regulatory network during OA progression.

Previous studies have identified WDR5 as a core subunit of the MLL/COMPASS H3K4 methyltransferase complex, which promotes H3K4me3 deposition and maintains chromatin accessibility and transcriptional activity [23]. To further validate whether WDR5 identified by exosomal proteomics serves as a functional cargo, we systematically examined its localization and delivery within exosomes. WB analysis confirmed a stable WDR5 signal in TGFβ-Exo-NP, alongside strong expression of canonical exosomal markers CD63 and TSG101, while the intracellular marker Calnexin was absent, indicating high exosome purity (Fig. S5E). A proteinase K digestion assay further demonstrated that the WDR5 signal remained unaffected in the absence of Triton X-100, but was significantly diminished upon Triton treatment (Fig. S5F), indicating that WDR5 is localized within the exosomal lumen.

### 3.13 WDR5-KD disrupts TGFβ-exo-NP-mediated mitochondrial homeostasis and impairs cartilage matrix repair capacity

**Fig. 10:**
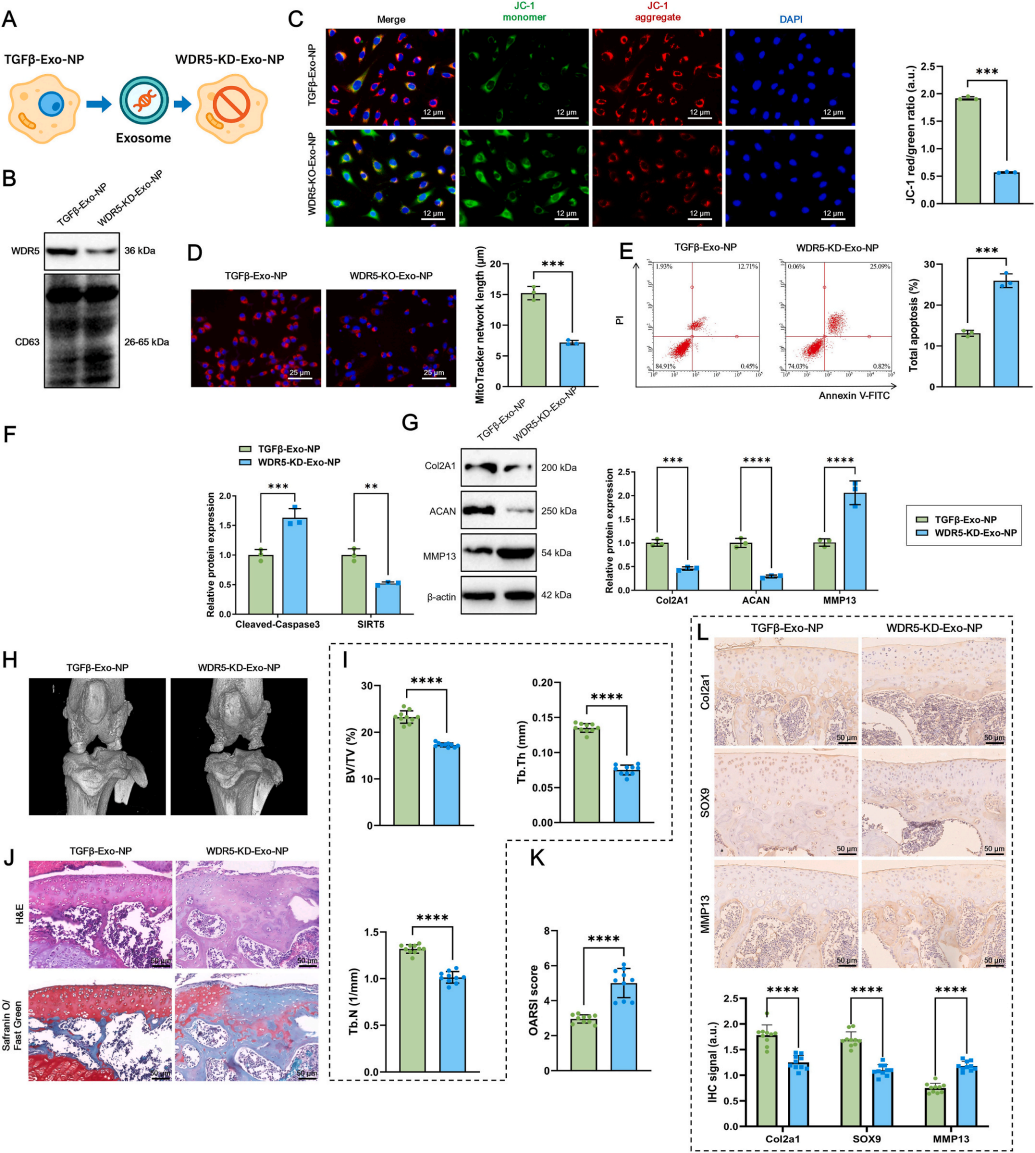
Impact of Functional Protein Depletion on Exosome Therapeutic Efficacy Note: (A) Schematic illustrating the generation of WDR5-KD-Exo-NP. (B) WB analysis of WDR5 protein expression in exosomes. (C) JC-1 fluorescence staining to assess changes in mitochondrial membrane potential. Scale bar = 12 μm. (D) MitoTracker Red staining to visualize mitochondrial morphology and distribution. Scale bar = 25 μm. (E) Annexin V/PI dual staining flow cytometry to determine the proportion of apoptotic cells. (F) WB analysis of cleaved caspase-3 and SIRT5 protein expression. (G) WB analysis of cartilage matrix-associated proteins Col2a1, Acan, and MMP13. (H) Three-dimensional micro-CT reconstruction was performed to examine the subchondral bone structure of the knee joint and to assess trabecular morphology and continuity; scale bar = 1 mm. (I) Quantitative analysis of BV/TV, Tb.Th, and Tb.N. (J-K) H&E staining and Safranin O/Fast Green histological staining were used to evaluate cartilage structure and OARSI scores; scale bar = 50 μm. (L) IHC analysis of Col2a1, SOX9, and MMP13 protein expression and distribution in cartilage tissue; scale bar = 50 μm. All cell-based experiments were independently repeated at least three times, and *in vivo* animal experiments included N = 10 animals per group. ***p < 0.001, ****p < 0.0001.

To elucidate the role of the key exosomal component WDR5 in the therapeutic effects of TGFβ-Exo-NP, we first validated its expression within exosomes and the efficiency of its knockdown by generating WDR5-KD exosomes (Fig. 10A). WB analysis revealed a marked reduction in WDR5 protein levels in the WDR5-KD-Exo-NP group compared to the TGFβ-Exo-NP group, indicating successful gene silencing and efficient production of WDR5-KD exosomes (Fig. 10B). Subsequently, JC-1 fluorescence staining was employed to assess changes in mitochondrial membrane potential. Chondrocytes treated with TGFβ-Exo-NP exhibited strong red fluorescence, indicative of well-maintained mitochondrial membrane potential. In contrast, cells treated with WDR5-KD-Exo-NP displayed markedly reduced red fluorescence and increased green fluorescence, with a significantly decreased red/green fluorescence ratio, suggesting that WDR5-KD compromised the protective effect of TGFβ-Exo-NP on mitochondrial polarization (Fig. 10C). Further analysis using MitoTracker Red fluorescence staining revealed that mitochondria in the TGFβ-Exo-NP group were uniformly distributed and exhibited an elongated, rod-like morphology. In contrast, the WDR5-KD-Exo-NP group showed fragmented, punctate, and disorganized mitochondrial structures, indicating that WDR5-KD exacerbated mitochondrial morphological damage (Fig. 10D).

Flow cytometry analysis further confirmed changes in apoptosis (Fig. 10E). Annexin V/PI double-staining revealed that the total apoptosis rate in the WDR5-KD-Exo-NP group was significantly elevated compared to the TGFβ-Exo-NP group. To further investigate the underlying molecular events, WB analysis was performed to assess the expression of cleaved caspase-3 and SIRT5. The results showed that cleaved caspase-3 levels were re-elevated, while SIRT5 expression declined in the WDR5-KD-Exo-NP group relative to the TGFβ-Exo-NP group (Fig. 10F).

In addition, to explore the role of WDR5 in maintaining cartilage matrix homeostasis, the protein expression levels of Col2a1, Acan, and MMP13 were evaluated (Fig. 10G). Compared with the TGFβ-Exo-NP group, the WDR5-KD-Exo-NP group exhibited reduced expression of Col2a1 and Acan, along with increased MMP13 levels, indicating that WDR5-KD impaired the matrix remodeling capacity of TGFβ-Exo-NP in chondrocytes.

The role of WDR5 in TGFβ-Exo-NP-mediated chondrocyte protection was further examined *in vivo*. Micro-CT analysis showed that rats in the TGFβ-Exo-NP group exhibited well-preserved subchondral bone architecture in the knee joint, characterized by densely packed trabeculae with good continuity, as well as relatively high levels of BV/TV, Tb.Th, and Tb.N. In contrast, the WDR5-KD-Exo-NP group displayed markedly sparse and disrupted trabecular structures, with pronounced reductions in trabecular continuity and density; BV/TV, Tb.Th, and Tb.N were all significantly lower than those observed in the TGFβ-Exo-NP group, indicating that WDR5 downregulation weakened the protective effects of exosomes on bone microarchitecture (Fig. 10H–I). Histological evaluation using Safranin O/Fast Green staining further corroborated this trend. In the TGFβ-Exo-NP group, the cartilage surface appeared smooth, chondrocytes were well organized, proteoglycan staining was intense, and OARSI scores were relatively low. By contrast, the WDR5-KD-Exo-NP group exhibited a rough cartilage surface, disordered cellular arrangement, markedly diminished matrix staining, and significantly elevated OARSI scores, indicating that WDR5 deficiency compromised the ability of exosomes to repair cartilage structure and maintain matrix homeostasis (Fig. 10J–K). IHC analysis further revealed molecular-level differences: strong and evenly distributed Col2a1 and SOX9 signals were detected in cartilage tissues from the TGFβ-Exo-NP group, whereas expression of both markers was substantially reduced in the WDR5-KD-Exo-NP group. Conversely, MMP13 expression was markedly increased in the WDR5-KD-Exo-NP group, suggesting enhanced matrix degradation activity (Fig. 10L).

Collectively, these findings demonstrate that WDR5 downregulation significantly attenuates the *in vivo* protective effects of TGFβ-Exo-NP on subchondral bone reconstruction, cartilage matrix synthesis, and suppression of matrix degradation, identifying WDR5 as a critical effector molecule underlying the reparative and anti-degenerative actions of TGFβ-Exo-NP.

## 4 Discussion

This study systematically elucidates, for the first time, that the TGFβ-Exo-NP exerts its therapeutic effects by activating the SOX3-H3K4me3-SIRT5 signaling axis, thereby coordinately regulating the epigenetic transcriptional program and mitochondrial homeostasis in chondrocytes. Through this mechanism, TGFβ-Exo-NP attenuates apoptosis-, pyroptosis-, and ferroptosis-related cell death signals and promotes the reconstruction of matrix metabolism, exhibiting robust chondroprotective and tissue-reparative effects both *in vitro* and *in vivo*. This work not only integrates two core pathological hallmarks of OA—epigenetic modification and metabolic reprogramming—but also introduces key innovations in exosome-based targeted delivery and the functional characterization of exosomal protein cargo. Compared with current OA treatments that primarily focus on symptom control [4], the findings presented here offer a feasible new strategy for disease-modifying therapy and establish both technological and theoretical foundations for the precision intervention of degenerative diseases.

In contrast to traditional OA therapies [24], TGFβ-Exo-NP demonstrates significant advancements in both therapeutic targeting and delivery methodology. Conventional medications, such as nonsteroidal anti-inflammatory drugs, hyaluronic acid, and corticosteroids, primarily alleviate pain or provide short-term functional relief, but are limited by modest long-term efficacy and associated side effects [25,26]. Although emerging stem cell therapies and biologics show promise, their clinical translation remains hindered by challenges in delivery efficiency, targeting precision, and immunogenicity [27]. As a novel class of nanoscale carriers, exosomes possess unique advantages—including endogenous origin, high biocompatibility, and engineering flexibility—making them ideal platforms for the delivery of therapeutics and bioactive molecules [[28], [29], [30]]. In this study, surface engineering was employed to enhance exosomal affinity toward TGF-βRs, enabling active targeting of chondrocytes within the inflammatory OA microenvironment. This modification markedly improved therapeutic selectivity and bioavailability while minimizing off-target effects commonly associated with non-specific delivery.

In terms of transcriptional regulatory mechanisms, this study is the first to identify SOX3 as a central modulator in OA, addressing a critical gap in the understanding of its physiological role. While the involvement of SOX family transcription factors such as SOX9 and SOX5 in chondrogenesis has been extensively documented [[31], [32], [33]]; however, the function and underlying mechanisms of SOX3 in OA chondrocytes remain insufficiently explored. We found that SOX3 expression is suppressed under inflammatory stimulation, whereas TGFβ-Exo-NP treatment markedly restored its activity. This restoration was accompanied by the recruitment of the H3K4 methyltransferase complex, thereby enhancing the transcription of nuclear protective genes and establishing a SOX3-H3K4me3 epigenetic activation axis. These findings not only broaden the functional scope of SOX3 in joint pathophysiology but also pave the way for epigenetic therapy in OA.

Metabolic reprogramming constitutes another critical mechanism underlying OA progression, with increasing evidence supporting the pivotal role of mitochondrial dysfunction in cartilage degradation. Although prior studies have shown that mitochondrial deacetylases such as SIRT1 and SIRT3 can partially improve chondrocyte energy metabolism [21,34], our study is the first to identify SIRT5 as a key downstream effector of the SOX3-H3K4me3 pathway involved in the restoration of mitochondrial function. We demonstrated that TGFβ-Exo-NP treatment significantly upregulated SIRT5 expression, improved mitochondrial membrane potential and ATP production, and mitigated oxidative stress, indicating a vital role for SIRT5 in maintaining mitochondrial homeostasis and cellular viability. Compared with single-target antioxidant or metabolic interventions, the epigenetic-metabolic coupling mechanism proposed in this study offers superior systemic regulatory capacity and therapeutic durability.

WDR5, a core component of the H3K4 methyltransferase complex, has been well established as a critical epigenetic regulator in cancer and stem cell research [20,35]. In this study, we identified WDR5 for the first time as a functional protein cargo within exosomes and demonstrated its essential role in mediating the protective effects of TGFβ-Exo-NP. Through knockdown experiments, we found that loss of WDR5 significantly impaired the activation of the SOX3-H3K4me3 axis, thereby downregulating SIRT5 expression, disrupting mitochondrial functional recovery, and compromising matrix synthesis. This finding offers a new paradigm for identifying functional protein cargos in exosomes, diverging from the conventional miRNA-centered approach and expanding the toolkit for targeted delivery strategies.

*In vivo* validation further confirmed the therapeutic potential of TGFβ-Exo-NP. The DMM rat model, a well-established OA model, provided a robust platform for disease simulation. We observed that TGFβ-Exo-NP exerted pronounced therapeutic effects across multiple dimensions, including subchondral bone reconstruction, proteoglycan preservation, reduced OARSI scores, and improved gait function. These effects were substantially attenuated following WDR5-KD, establishing a direct mechanistic-functional relationship. In contrast to previous studies, which often lack integration between mechanistic investigation and functional validation, our study combines target-specific interference with phenotypic assessment, thereby enhancing the credibility of the results and their potential for clinical translation.

Moreover, compared with the currently prevalent mesenchymal stem cell (MSC)-derived exosomes [36,37], the chondrocyte-derived exosomes used in this study exhibit superior cellular compatibility and tissue-specific recognition. These exosomes demonstrate distinct advantages in targeted delivery, cargo selectivity, and functional stability. In addition, by integrating engineering modifications, multi-omics profiling, and *in vivo* validation, this study constructed a comprehensive OA intervention framework with both theoretical depth and technical breadth, thereby supplementing and extending the current scope of exosome-based research in joint diseases.

In summary, this study systematically elucidated the mechanism by which TGFβ-Exo-NP reverses OA pathology by activating the SOX3-H3K4me3-SIRT5 signaling axis, highlighting the pivotal regulatory role of WDR5 as a functional cargo within this coordinated network. The work presents notable innovations in the design of delivery systems, epigenetic regulatory mechanisms, and the analysis of functional proteins in exosomes. Scientifically, it establishes a novel paradigm of epigenetic-metabolic coupling regulation, expanding the molecular foundation of OA pathogenesis. Clinically, the strategy offers promising biocompatibility, target specificity, and translational potential, making it a viable candidate for future disease-modifying therapies. Nonetheless, certain limitations remain: long-term toxicity assessments are pending, the therapeutic window and dose-response relationships require further clarification, and validation in human OA tissues is still lacking. Future research should focus on multicenter preclinical studies, optimization of delivery systems, and combinatorial applications of diverse exosomal cargos to facilitate the translation of this technology from bench to bedside.

## Ethical statement

All animal experiments were approved by the Animal Ethics Committee of Shanghai Changzheng Hospital.

## Funding

None.

## CRediT authorship contribution statement

**Fu Qiwei:** Conceptualization, Data curation, Formal analysis, Investigation, Methodology, Validation, Visualization, Writing – original draft. **Shao Jiahua:** Data curation, Formal analysis, Investigation, Methodology, Validation, Visualization. **Chen Shu:** Data curation, Formal analysis, Methodology, Validation, Visualization. **Cao Jia:** Conceptualization, Funding acquisition, Software, Writing – review & editing. **Li Haobo:** Conceptualization, Project administration, Supervision, Writing – review & editing. **Zhu Jun:** Conceptualization, Project administration, Supervision, Writing – review & editing.

## Declaration of competing interest

The authors declare the following financial interests/personal relationships which may be considered as potential competing interestsThe authors declare that they have no known competing financial interests or personal relationships that could have appeared to influence the work reported in this paper.

## Data Availability

Data will be made available on request.

## Abbreviations

ANOVA: Analysis of Variance
ATP: Adenosine Triphosphate
BCA: Bicinchoninic Acid
BSA: Bovine Serum Albumin
BV/TV: Bone Volume Fraction
ChIP-qPCR: Chromatin Immunoprecipitation Followed by Quantitative Polymerase Chain Reaction
ChIP-seq: Chromatin Immunoprecipitation Sequencing
DEGs: Differentially Expressed Genes
DEPs: Differentially Expressed Proteins
DLS: Dynamic Light Scattering
DMM: Destabilization of the Medial Meniscus
ECL: Enhanced Chemiluminescence
FBS: Fetal Bovine Serum
FDR: False Discovery Rate
GAG: Glycosaminoglycan
GO: Gene Ontology
H&E: Hematoxylin and Eosin
IHC: Immunohistochemistry
KEGG: Kyoto Encyclopedia of Genes and Genomes
LC-MS/MS: Liquid Chromatography-Tandem Mass Spectrometry
LNP: Lipid Nanoparticle
Mean ± SD: Mean ± Standard Deviation
MFI: Mean Fluorescence Intensity
Micro-CT: Micro-Computed Tomography
MOI: Multiplicity of Infection
NTA: Nanoparticle Tracking Analysis
OA: Osteoarthritis
PBS: Phosphate-Buffered Saline
PCA: Principal Component Analysis
PDI: Polydispersity Index
PI: Propidium Iodide
PLGA: Poly(Lactic-Co-Glycolic Acid)
PLS-DA: Partial Least Squares Discriminant Analysis
PPI: Protein-Protein Interaction
QC: Quality Control
qPCR: Quantitative Polymerase Chain Reaction
ROI: Region of Interest
ROS: Reactive Oxygen Species
RNA-seq: RNA Sequencing
RT-qPCR: Reverse Transcription Quantitative Polymerase Chain Reaction
SD: Sprague-Dawley
shSOX3: SOX3 Knockdown
SPF: Specific Pathogen-Free
Tb.N: Trabecular Number
Tb.Th: Trabecular Thickness
TCA: Tricarboxylic Acid
TEM: Transmission Electron Microscopy
TGFβ-Exo-NP: Transforming Growth Factor-β Receptor-Targeted Chondrocyte-Derived Exosome-Nanoparticle Complex
TGF-βR: Transforming Growth Factor-β Receptor
VIP: Variable Importance in Projection
WB: Western Blot
WDR5-KD: WDR5 Knockdown
WDR5-KD-Exo-NP: WDR5 Knockdown Chondrocyte-Derived Exosome-Nanoparticle Complex
