# Supplementary material for: Epigenetic–metabolic coupling by SOX3/WDR5-SIRT5 signaling mediates the therapeutic effects of TGFβ-targeted exosomal nanoparticles in osteoarthritis
Source: Mater Today Bio. 2026 Jul 21;39:103457. doi: 10.1016/j.mtbio.2026.103457 (PMC13449358; doi:10.1016/j.mtbio.2026.103457)
Supplement: Supplementary file 1 [file mmc1.docx]

**Figure S1. Multi-Omics Integration Reveals the SOX3-H3K4me3-Linked Cell Death-Related Regulatory Network of TGFβ-Exo-NP.**

Note: (A) Volcano plot of DEGs from RNA-seq analysis, N = 3. (B) GO enrichment analysis of DEGs. (C) KEGG pathway network analysis of DEGs. (D) Volcano plot of DEPs from TMT-based proteomics, N = 5. (E) Heatmap of DEP profiles. (F) GO enrichment analysis of DEPs. (G) KEGG pathway enrichment of DEPs. (H) ChIP-seq analysis showing the H3K4me3 peak distribution at the Sox3 promoter region; peak calling was performed using MACS2.

**Figure S2. Untargeted Metabolomics and Cellular Energy Metabolism Assessment.**

Note: (A) Volcano plot showing the distribution of differentially expressed metabolites identified by untargeted LC-MS (N = 6). (B) PLS-DA plot distinguishing metabolic profiles between groups. (C) VIP ranking plot identifying key metabolites based on VIP scores from the PLS-DA model. (D) Heatmap of differentially expressed metabolites based on hierarchical clustering of metabolite abundance. (E) KEGG-based pathway network illustrating the relationships among significantly altered metabolites. (F) Lactate concentration was quantified using a colorimetric lactate assay kit. (G) The NAD^+^/NADH ratio is measured to assess cellular redox status. All experiments were conducted in triplicate or more. ***p <* 0.01, ****p <* 0.001.

**Figure S3. TGFβ-Targeted Exosome Nanoparticles Significantly Improve Gait Behavior and Motor Dysfunction in the DMM Model.**

Note: (A) Three-dimensional micro-CT reconstruction was used to evaluate changes in joint structure, illustrating the status of the meniscus and joint cavity; scale bar = 1 mm. (B-D) Gait analysis assessing stride width, stride length, and toe spread. (E) Measurement of knee joint circumference to evaluate the degree of joint swelling. (F) Forelimb grip strength testing to assess muscle strength and load-bearing capacity. (G-J) Gait analysis evaluating stride width, stride length, and toe spread. (K) Forelimb grip strength assessment using a grip strength meter to measure maximal forelimb force. (L) Schematic illustration demonstrating that TGFβ-Exo-NP exhibits efficient *in vivo* delivery and cartilage reparative effects, which are dependent on SOX3-mediated activation. N = 10. ***p* < 0.01, ****p* < 0.001, *****p* < 0.0001.

**Figure S4. TGFβ-Exo-NP did not induce evident organ toxicity.**

Note: (A) H&E staining showed that TGFβ-Exo-NP did not cause obvious histopathological changes in the heart, liver, spleen, lung, or kidney; scale bar = 50 μm. (B) Serum biochemical analysis showed that TGFβ-Exo-NP did not alter alanine aminotransferase (ALT), aspartate aminotransferase (AST), blood urea nitrogen (BUN), or creatinine (Cr) levels. n = 10.

**Figure S5. Differential Protein Screening, Enrichment Pathway Analysis, and WDR5 Validation.**

Note: (A) Volcano plot of DEPs (N = 5). (B) Heatmap showing the expression profiles of differential proteins. (C) GO enrichment analysis of DEPs. (D) KEGG pathway enrichment analysis. (E) WB analysis of WDR5, CD63, TSG101, and Calnexin expression. (F) Quantification of WDR5 protein levels. All experiments were independently repeated at least three times. ns: not significant; **p <* 0.05, *****p <* 0.0001.
